# MYCN inhibits TrkC-mediated differentiation in neuroblastoma cells via disruption of the PKA signalling pathway

**DOI:** 10.1038/s41420-026-03024-y

**Published:** 2026-03-25

**Authors:** Stephanie Maher, Andrew Roe, Kieran Wynne, Vadim Zhernovkov, Melinda Halasz

**Affiliations:** 1 Systems Biology Ireland, School of Medicine, University College Dublin, Belfield, Ireland; 2 Conway Institute of Biomolecular and Biomedical Research, University College Dublin, Belfield, Ireland

**Keywords:** Paediatric cancer, Paediatric cancer, Oncogenesis

## Abstract

Neuroblastoma is a rare childhood cancer in which high-risk disease, frequently driven by MYCN amplification, has poor survival. Trk-receptor expression correlates with prognosis: TrkA is observed in low-risk cases while TrkB is often expressed in high-risk MYCN-amplified neuroblastoma. However, TrkC’s role in neuroblastoma genesis remains unclear. This study investigates the interplay between TrkC signalling and MYCN status. Using neuroblastoma cell lines with varying MYCN levels, we found that TrkC activation leads to neuronal differentiation in MYCN non-amplified cells but promotes proliferation in MYCN-overexpressing and MYCN-amplified cells. Temporal phosphoproteomic analysis identified the PKA pathway as crucial for TrkC-mediated differentiation. Manipulating PKA signalling altered cell fate in vitro and in zebrafish xenografts. In MYCN-amplified cells, MYCN knockdown enhanced PKA/CREB signalling and induced differentiation. Similarly, overexpression of constitutively active PKA or CREB promoted differentiation, confirming the role of PKA/CREB pathway in driving differentiation. Analysis of patient data revealed reduced expression of PKA pathway genes in MYCN-amplified tumours. Additionally, MYCN-induced miR-221 was found to suppress CREB expression. Together, these findings demonstrate MYCN-dependent effects of TrkC signalling and highlight the therapeutic potential of targeting the PKA pathway to induce differentiation in high-risk MYCN-amplified neuroblastoma.

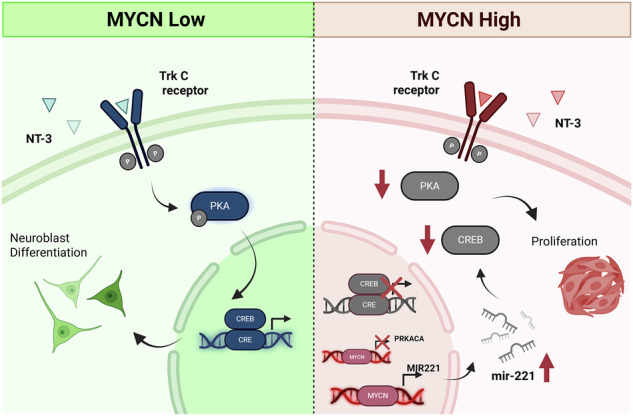

## Introduction

Neuroblastoma is a paediatric malignancy arising from neural crest cells of the developing sympathetic nervous system. Extreme clinical heterogeneity is a hallmark of neuroblastoma, ranging from spontaneous tumour regression to aggressive metastatic disease [1, 2]. Patients are stratified into low-, intermediate- and high-risk groups, defined by prognostic factors including age at diagnosis, tumour stage, histological features, and cytogenetic findings [3, 4]. Low-risk patients are typically managed with observation or surgical resection of the primary tumour, achieving 5-year overall survival rates of over 90%. Some of these tumours may spontaneously regress through apoptosis or neuroblast differentiation [5]. In contrast, approximately half of the patients are classified as high-risk and harbour aggressive, metastatic neuroblastomas that often resist treatment and subsequently relapse. Despite intensive multimodal therapy, the 5-year overall survival in this group remains below 50% [3, 5].

The neurotrophic tyrosine receptor kinase (NTRK) family, comprising TrkA, TrkB, and TrkC (gene names: NTRK1, NTRK2, and NTRK3), as well as their respective ligands NGF, BDNF, and NT-3, are differentially expressed in the divergent clinical phenotypes observed in neuroblastoma patients [6–9]. TrkA expression correlates with low-stage disease and tumours that undergo spontaneous regression [6, 10, 11]. Conversely, TrkB is predominantly expressed in aggressive, high-risk neuroblastoma, and promotes angiogenesis, proliferation, invasion, and therapy resistance [12–15].

Compared with TrkA and TrkB, the role of TrkC in neuroblastoma remains less well defined. Several studies have reported high TrkC expression in low-stage tumours with favourable prognosis, often co-expressed with TrkA [8, 16, 17]. However, TrkC has also been implicated in oncogenic signalling, as a subset of advanced stage 4 neuroblastomas exhibits co-expression of TrkC and its ligand NT-3, suggesting the presence of an autocrine survival and proliferation loop [18, 19]. Beyond neuroblastoma, the role of TrkC in cancer is multifaceted. In some cancers, such as medulloblastoma and colon cancer, TrkC expression is associated with favourable outcomes, suggesting a potential tumour-suppressive function [20–22]. However, the opposite is observed in breast cancer and leukaemia, where TrkC promotes oncogenic signalling [23, 24]. This duality underscores the context-dependent nature of TrkC signalling, indicating that TrkC’s effects on cell behaviour are highly influenced by the cellular and molecular environment in which it operates.

The MYCN oncogene plays an integral role in neuroblastoma development. During normal embryogenesis, MYCN is transiently expressed in the ventrolateral migrating cells of the neural crest that give rise to the sympathetic ganglia, mediating cellular proliferation, migration, and differentiation through the activation and repression of target genes [25, 26]. MYCN amplification occurs in approximately 20-25% of neuroblastomas and independently defines a high-risk diagnosis. These tumours exhibit a highly aggressive phenotype and, compared to MYCN non-amplified neuroblastoma, a considerably worse outcome [27–32]. In addition to promoting proliferation, MYCN amplification enhances angiogenesis, metastasis and evasion of immune surveillance [26, 33–36]. It also potently inhibits differentiation pathways and maintains self-renewal and pluripotency, creating a highly aggressive tumour microenvironment. Notably, MYCN amplification is detected in 30-40% of stage 3 and 4 neuroblastoma patients [37].

Currently, the interplay between MYCN status and NT-3/TrkC signalling in neuroblastoma remains poorly understood. The dual role of TrkC, acting as either an oncogene or a tumour suppressor depending on the cellular context, highlights the need for precise molecular characterisation of its signalling mechanisms. This study aims to elucidate the dynamics of the NT-3/TrkC signalling network and its impact on cell fate decisions in neuroblastoma, with a particular focus on how the oncogenic driver MYCN shapes this signalling landscape.

## Results

### NT-3/TrkC signalling directs different cell fates in neuroblastoma cells with varying MYCN levels

**Fig. 1:**
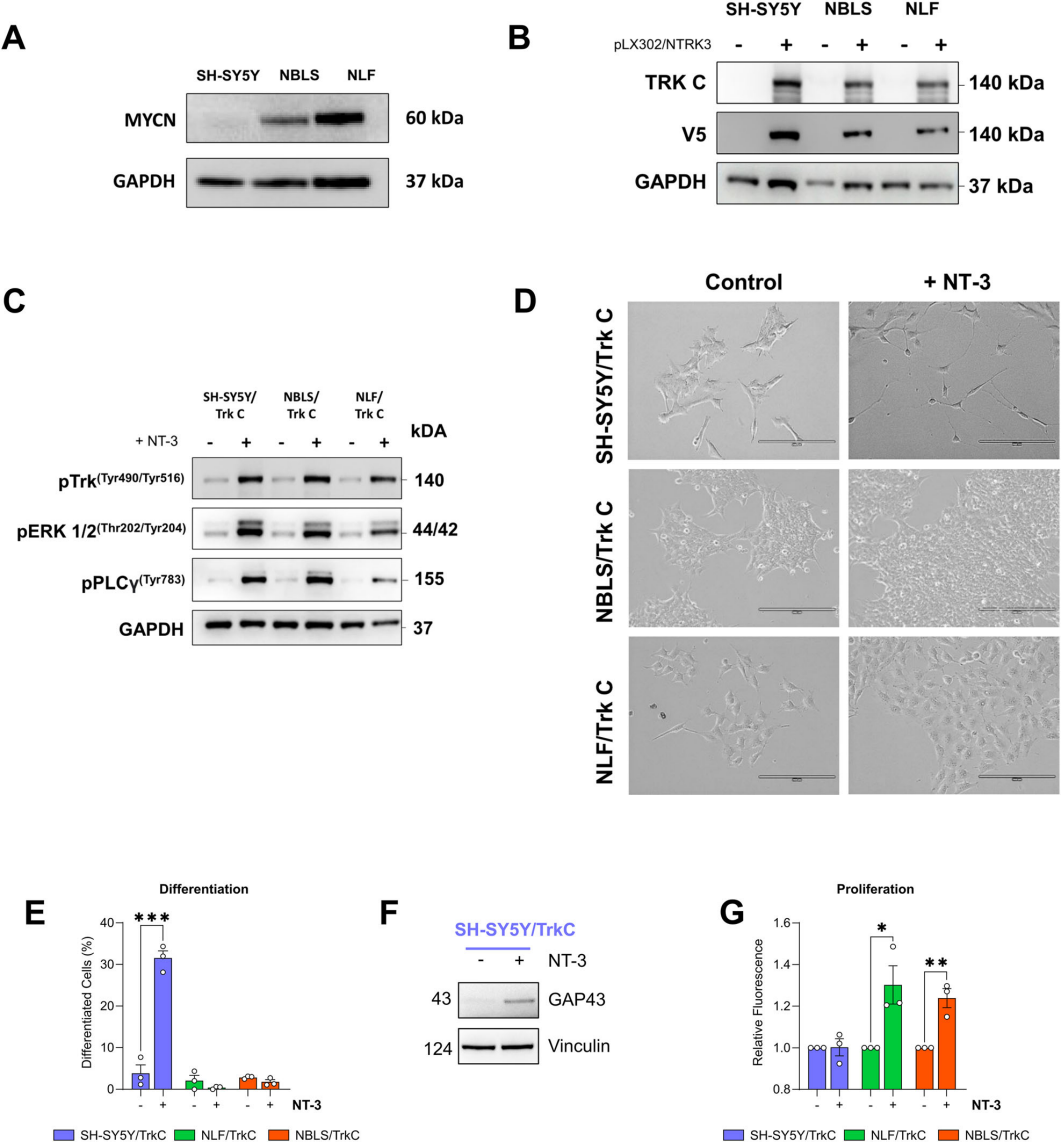
TrkC drives divergent cellular phenotypes in neuroblastoma cells with different MYCN levels. **A** MYCN expression of the SH-SY5Y, NBLS and NLF parental neuroblastoma cell lines by Western blotting. **B** TrkC and V5-tag expression in parental neuroblastoma cell lines and pLX302/NTRK3 transfected cells. **C** Western blot analyses of phospho-TrkC (Tyr^516^), phospho-ERK (Thr^202^/Tyr^204^), phospho-PLCγ (Tyr^783^) following stimulation of SH-SY5Y/TrkC, NBLS/TrkC, NLF/TrkC cells with 100 ng/ml of NT-3 for 10 min. GAPDH acted as loading control for Western blot experiments. **D** Representative images of phenotypic observation by light microscopy 5 days post NT-3 (100 ng/ml) stimulation in SH-SY5Y/TrkC, NLF/TrkC and NBLS/TrkC neuroblastoma cells. Magnification: X20; scalebar: 200 µm. **E** Quantification of neuronal differentiation following NT-3 (100 ng/ml) treatment for 5 days. (*n* = 3). **F** Western blot analysis of GAP43, a neuronal differentiation marker, following stimulation of SH-SY5Y/TrkC cells with 100 ng/ml of NT-3 for 5 days. Vinculin acted as loading control. (*n* = 3). **G** Quantification of mean fluorescence as a measure of cell proliferation at 72 h post NT-3 (100 ng/ml) stimulation using CyQuant assay. Data shown as mean ± SEM; *n* = 3 (*p* < 0.05 = *, *p* < 0.001 = **, *p* < 0.0001 = ***).

To investigate TrkC receptor signalling in neuroblastoma, we first established a panel of cell lines stably expressing the NTRK3 gene, which encodes the TrkC protein. We chose the SH-SY5Y, NBLS, and NLF neuroblastoma cell lines (Table 1) for transfection, based on their differing MYCN statuses (Fig. 1A) and lack of basal TrkC expression (Fig. 1B). SH-SY5Y cells are MYCN non-amplified, NBLS cells overexpress MYCN from a single gene copy, and NLF cells harbour MYCN amplification. In addition, the MYCN expression levels are comparable in NBLS and NLF cells (Fig. 1A). Given that MYCN is the main oncogenic driver in neuroblastoma and its amplification is an independent prognostic marker of high-risk disease, this panel of cell lines with varying MYCN levels enables investigation of how MYCN status influences TrkC signalling.

**Table 1:** Genetic profiles of the neuroblastoma cell lines used in the project [57].

Cell line	*MYCN* status	1p36 del	3p26 del	11q23 del	17q unbalanced gain	*ALK* mutation	*TP53* mutation
SH-SY5Y	Non-amplified	−	−	+	+	F1174L	WT
NBL-S	Non-amplified, MYCN overexpression	−	−	+	+	WT	WT
NLF	Amplified	+	+	+	+	WT	V203M
IMR-32	Amplified	+	+	+	+	WT	WT

Stimulation of TrkC-expressing neuroblastoma cells with its cognate ligand, NT-3, resulted in receptor phosphorylation and activation of downstream pathways, including ERK and PLCγ (Fig. 1C). This response is consistent with canonical Trk receptor signalling and aligns with previous studies, supporting the suitability of our system to study TrkC signalling in neuroblastoma [38–40].

To investigate cell fate decisions mediated by TrkC signalling, we assessed neuronal differentiation and proliferation following NT-3 treatment. These are the two predominant cell fates in neuroblast development, correlating clinically with spontaneous regression and aggressive metastatic progression, respectively. Interestingly, cell fate decisions directed by NT-3/TrkC signalling were different in the three cell lines. NT-3 stimulation of SH-SY5Y/TrkC cells induced neuronal differentiation without a significant change in cell number compared to the untreated control (Fig. 1D–G). In contrast, the MYCN-overexpressing NBLS/TrkC and MYCN-amplified NLF/TrkC cells failed to undergo differentiation and instead exhibited a significant increase in proliferation upon NT-3 treatment. Moreover, we also assessed the expression of neuronal differentiation markers at the protein level. The increased expression of ELAVL4 [41] at 24 h and GAP43 at 5 days of NT-3 treatment further confirms that NT-3 treated SH-SY5Y/TrkC cells undergo neuronal differentiation (Figure S1 and Fig. 1F).

These findings highlight a MYCN-dependent divergence in NT-3/TrkC-mediated cell fate decisions in neuroblastoma, suggesting that the distinct cell fates observed might be driven by differences in MYCN expression levels.

### Temporal phosphoproteomics of NT-3/TrkC signalling reveals differential PKA pathway activation across neuroblastoma cell lines

**Fig. 2:**
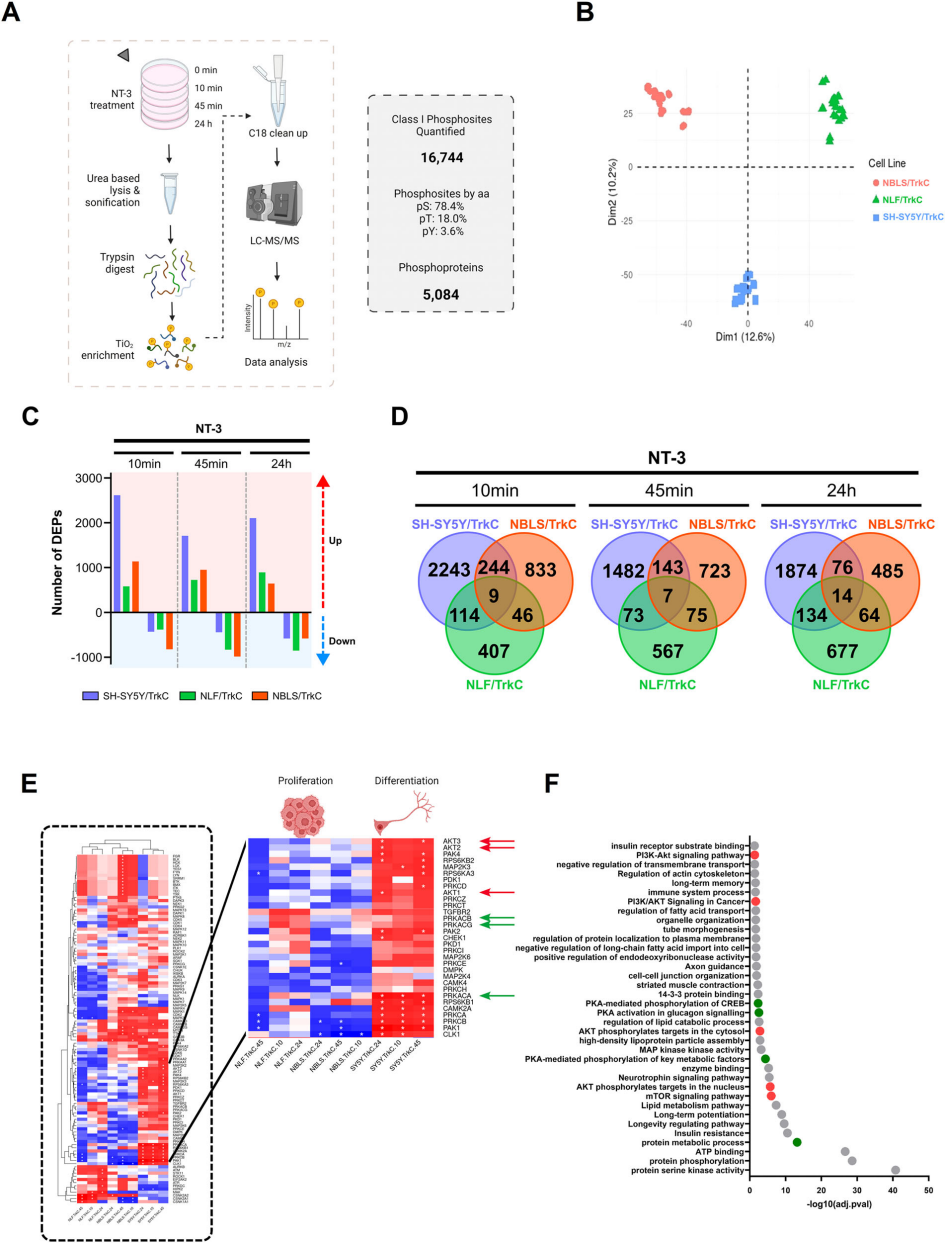
Phosphoproteomic analysis reveals differences in NT-3/TrkC signalling across SH-SY5Y/TrkC, NBLS/TrkC and NLF/TrkC cells. **A** Quantitative phosphoproteomics workflow for analysing NT-3/TrkC signalling in neuroblastoma cells, and summary of phosphoproteome data, including the number of identified phosphorylation sites and phosphoproteins (3 cell lines, 4 time points, 3 biological and 2 technical replicates resulting in 72 MS runs). **B** Principal component analysis (PCA) plot of all phosphoproteomic samples (*n* = 72). **C** Number of upregulated and downregulated differentially expressed phosphosites (DEPs) after 10, 45 min or 24 h of NT-3 treatment (adjusted *p* value < 0.05 and absolute fold change > 1.5). **D** Venn diagram of overlapping DEPs between each cell line at 10, 45 min and 24 h of NT-3 treatment. **E** Kinase substrate enrichment analysis with KSEAapp of DEPs in all samples (FDR < 0.05, m.cutoff = 3). **F** Pathway enrichment analysis [82] for all upregulated DEPs in SH-SY5Y/TrkC cells, *p* value < 0.05.

To understand the dynamic changes in the NT-3/TrkC downstream signalling network that may underlie the divergent cell fate decisions observed, we employed a quantitative phosphoproteomic approach. Temporal profiling of SH-SY5Y/TrkC, NBLS/TrkC, and NLF/TRKC cells was carried out by stimulating the cells with NT-3 for 0, 10, 45 min and 24 h (Fig. 2A). This range of timepoints facilitated the capture of early, intermediate, and late phosphorylation events.

We identified 25098 phosphorylated sites, 16744 of which were confidently localized to serine (78.4% of the total), threonine (18.0%), or tyrosine (3.6%) residues in the peptide sequence class I with high reproducibility across replicates. Principle component analysis (PCA) of all phosphoproteomics samples revealed distinct clustering based on the parental cell line (Fig. 2B). Furthermore, assessment of differentially expressed phosphosites (DEPs) showed that SH-SY5Y/TrkC cells exhibit the highest number of upregulated DEPs compared to the other cell lines at each timepoint. NBLS/TrkC cells demonstrated the highest number of significantly downregulated phosphosites at 10 and 45 min of NT-3 treatment, while NLF/TrkC cells had the highest number of downregulated phosphosites after 24 h of NT-3 stimulation (Fig. 2C). Moreover, SH-SY5Y/TrkC cells also displayed the greatest number of unique DEPs at each timepoint of NT-3 treatment when comparing the overlapping DEPs (Fig. 2D). Taken together, the trends in the phosphoproteomic data correspond with the observed cell fate decisions. SH-SY5Y/TrkC cells demonstrate a strong positive response to NT-3 stimulation and harbour a distinctive phosphoproteome compared to NBLS/TrkC and NLF/TrkC cells. This enhanced response may be attributed to the known role of MYCN as a global suppressor of cell signalling in neuroblastoma [42], with signalling pathways predominantly activated in MYCN non-amplified SY5Y/TrkC cells but inhibited in MYCN-overexpressing NBLS/TrkC and MYCN-amplified NLF/TrkC cells.

As phosphoproteomic datasets can be large and complex to delineate, kinase substrate enrichment analysis (KSEA) was employed to focus on the most relevant phosphorylation changes in the NT-3/TrkC phosphoproteome data by linking the phosphorylation changes to key regulatory kinases, thus reducing data complexity.

Firstly, KSEA analysis showed kinase clustering according to whether cells undergo differentiation (SH-SY5Y/TrkC) or proliferation (NBLS/TrkC and NLF/TrkC) upon NT-3 stimulation, suggesting that downstream signalling networks mediate the distinct cell fate decisions. Protein Kinase A (PKA) and Protein Kinase B (AKT) were identified as key regulators of SH-SY5Y/TrkC signalling that were not enriched in NBLS/TrkC and NLF/TrkC cells (Fig. 2E).

Furthermore, pathway enrichment analysis of phosphoprotein kinases with increased activity in SH-SY5Y/TrkC cells showed several expected pathways including “neurotrophin signalling pathway”, “protein phosphorylation” and “protein serine kinase activity” (Fig. 2F). It also showed enrichment for processes relating to neuronal differentiation including “axon guidance”, “regulation of actin cytoskeleton” and “long term potentiation”. There were also several pathways relating to metabolism including “regulation of fatty acid transport”, “regulation of lipid catabolic process”, “protein metabolic process”, “lipid metabolism pathway”. Most notably, there was an enrichment for terms relating to the PKA and AKT pathways including “PI3K-Akt signalling pathway”, “AKT phosphorylation targets in the cytosol”, “mTOR signalling pathway” and “PKA-mediated phosphorylation of CREB”, “PKA activation in glucagon signalling”, “PKA-mediated phosphorylation of key metabolic factors”, further suggesting these pathways to play a role in NT-3/TrkC-mediated neuronal differentiation of SH-SY5Y/TrkC cells.

### Manipulation of the PKA pathway alters TrkC-mediated cell fate decisions in neuroblastoma

**Fig. 3:**
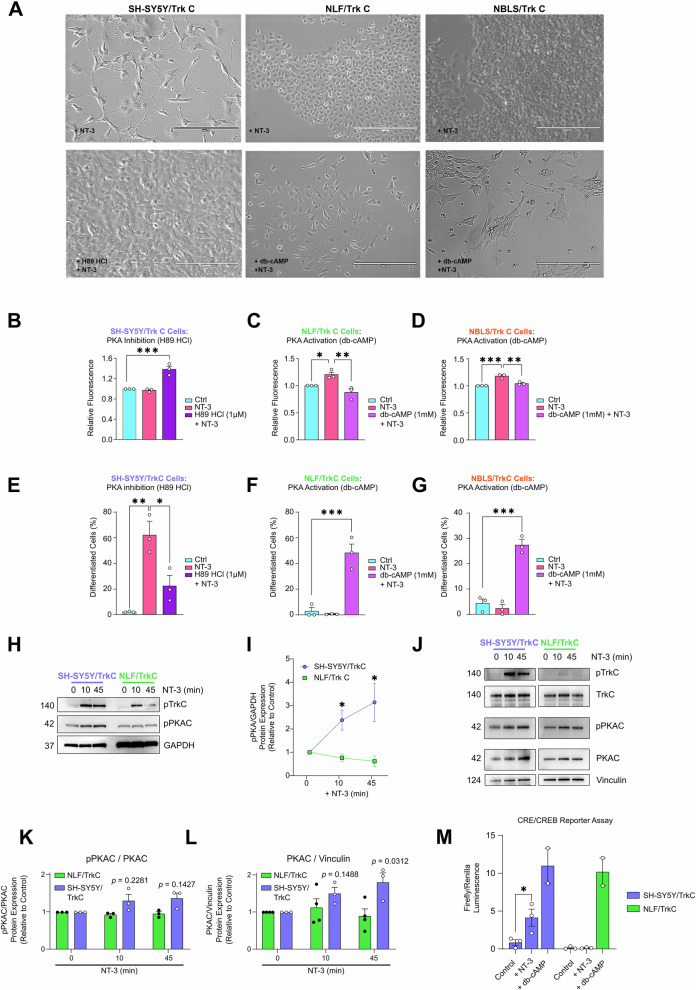
Manipulation of the PKA pathway alters TrkC-mediated cell fate decisions in neuroblastoma cells. **A** Representative images of cells following 5-day treatment with NT-3 (100 ng/ml) and/or H89 2HCl (1 μM) or db-cAMP (1 mM) in SH-SY5Y/TrkC, NLF/TrkC and NBLS/TrkC cells. Magnification: X20; scalebar: 200 µm. **B** Measure of cell proliferation using CyQuant assay following 72 h of treatment as in (**A**) in SH-SY5Y/TrkC, **C** NLF/TrkC and **D** NBLS/TrkC cells. Data was quantified as mean fluorescence as a measure of relative cell number. **E** Percentage of differentiated neurons following 5 days of treatment as in (A) in SH-SY5Y/TrkC, **F** NLF/TrkC and **G** NBLS/TrkC cells (*n* = 3). **H**–**L** Western blot analyses of phospho-TrkC (Tyr^516^), TrkC, phospho-PKA C (Thr^197^), PKA C-α in SH-SY5Y/TrkC and NLF/TrkC cells following 0, 10, 45 min treatment with NT-3 (100 ng/ml). GAPDH or vinculin acted as loading control (*n* = 3). **M** Measure of CRE/CREB transcriptional activity by luciferase reporter assay following NT-3 stimulation (100 ng/ml) in SH-SY5Y/TrkC and NLF/TrkC cells; db-cAMP (1 mM) was used as positive control. Data shown as mean ± SEM; *n* = 3 (*p* < 0.05 = *).

To test if the differential PKA signalling drives the different cell fate decisions observed, we inhibited or activated PKA signalling in SH-SY5Y/TrkC or NBLS/TrkC and NLF/TrkC cells, respectively. Pharmacological inhibition of PKA signalling by H89 dihydrochloride (H89 2HCl) resulted in increased cell number and attenuation of neuronal differentiation in NT-3 treated SH-SY5Y/TrkC cells (Fig. 3A, B, E). Conversely, activation of the PKA pathway in NT-3 treated NBLS/TrkC and NLF/TrkC cells by dibutyryl cyclic AMP (db-cAMP, a cell-permeable cAMP analogue), resulted in neuronal differentiation and inhibition of cell proliferation (Fig. 3A, C, D, F-G), highlighting a role for the PKA pathway in steering TrkC-mediated neuroblast cell fate decisions.

In its inactive state, PKA exists as a tetrameric holoenzyme composed of two catalytic (C) subunits (three isoforms: C-α, C-β, C-γ) and two regulatory (R) subunits (four isoforms: RI-α, RI-β, RII-α, RII-β) [43]. Activation results in a conformational change that causes the release and activation of catalytic subunits from the regulatory subunits. On assessment of signalling, PKA C protein levels increased and became phosphorylated at Thr^197^ upon NT-3 stimulation in SH-SY5Y/TrkC cells (Fig. 3H-L). In contrast, PKA C demonstrated no significant change in Thr^197^ phosphorylation in NLF/TrkC cells when stimulated with NT-3.

A putative event in PKA C downstream signalling is activation of the cAMP response element-binding protein (CREB), and initiation of transcription at target genes containing cAMP response elements (CREs) in their promoter regions. Evaluation of CRE/CREB transcriptional factor activity by luciferase reporter assay showed an increase in CRE/CREB transcriptional activity upon NT-3 stimulation in SH-SY5Y/TrkC cells, whereas no increase in NLF/TrkC cells compared to untreated control (Fig. 3M), thus confirming differences in the regulation of this pathway across cell lines.

### MYCN status influences TrkC downstream signalling and cell fate decisions in neuroblastoma cells

**Fig. 4:**
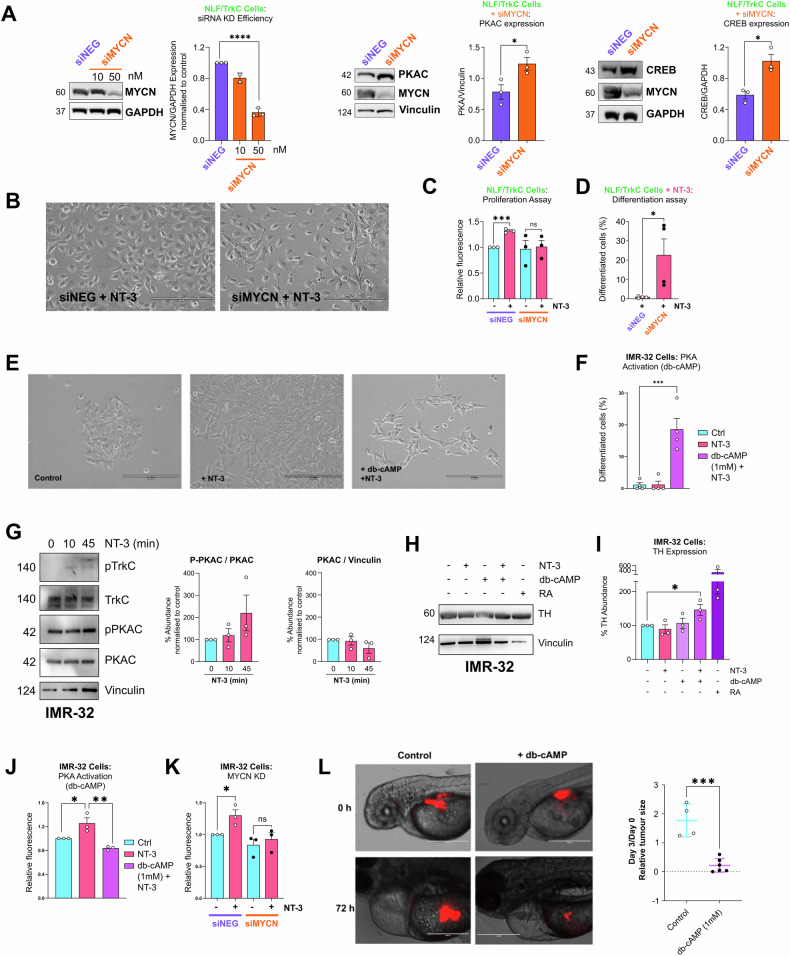
Knockdown of MYCN as well as reactivation of the PKA pathway in MYCN-amplified cells leads to neuronal differentiation. **A** Western blot analyses of MYCN, PKA C-α and CREB protein expression following siRNA-mediated knockdown of MYCN (siMYCN; 50 nM) or non-targeting control (siNEG) for 24 h in NLF/TrkC cells. Vinculin or GAPDH acted as loading control. Data presented as relative protein expression compared to loading control (mean ± SEM, *n* = 3). **B** Representative images of phenotypic observation by light microscopy after 5 days of siRNA-mediated knockdown of MYCN or non-targeting control in NLF/TrkC cells. Magnification: X20; scalebar: 200 µm. **C** Quantification of mean fluorescence as a measure of cell proliferation at 72 h post NT-3 (100 ng/ml) stimulation using CyQuant assay in NLF/TrkC cells with siRNA-mediated knockdown of MYCN or non-targeting control (*n* = 3). **D** Quantification of neuronal differentiation with NeuronJ following NT-3 (100 ng/ml) treatment for 5 days in NLF/TrkC cells with siRNA-mediated knockdown of MYCN or non-targeting control (*n* = 3). **E** Phenotypic observation by light microscopy after 5 days of treatment with NT-3 (100 ng/ml) and/or db-cAMP (1 mM) in IMR-32 cells. Magnification: X20; scalebar: 200 µm. **F** Quantification of neuronal differentiation with NeuronJ following NT-3 (100 ng/ml) and/or db-cAMP (1 mM) treatment for 4–5 days in IMR-32 cells (*n* = 3). **G** Western blot analyses of phospho-TrkC (Tyr^516^), TrkC, phospho-PKA C (Thr^197^), and PKA C-α in IMR-32 cells following 0, 10, 45 min stimulation with NT-3 (100 ng/ml). Vinculin acted as loading control (*n* = 3). **H**, **I** Western blot analysis of TH expression in IMR-32 cells treated with NT-3 (100 ng/ml) and/or db-cAMP (1 mM) or RA (10 μM) for 5 days. Vinculin acted as loading control (*n* = 3). **J** Quantification of mean fluorescence as a measure of cell proliferation at 72 h post NT-3 (100 ng/ml) stimulation and/or db-cAMP (1 mM) treatment using CyQuant assay (*n* = 3) or **K** following siRNA-mediated knockdown of MYCN or non-targeting control in IMR-32 cells. Data shown as mean ± SEM, *n* = 3 (*p* < 0.05 = *, *p* < 0.001 = **, *p* < 0.0001 = ***). **L** Representative images of tumour progression in non-treated and db-cAMP (1 mM) treated zebrafish. Scalebar: 400 µm. Graph shows relative tumour fluorescence compared to control at 72 h post db-cAMP treatment. Data represented as mean ± SEM (unpaired *t*-test, *p* < 0.001 = ***).

To ascertain whether differences in cell fate decisions and activation of the cAMP/PKA/CREB pathway between SH-SY5Y/TrkC cells and NLF/TrkC cells was primarily attributed to the difference in MYCN status rather than other genomic characteristics, siRNA-mediated knockdown (KD) of MYCN was employed. Following 24 h, MYCN protein expression levels were reduced by ~60% in NLF/TrkC cells (Fig. 4A). Consequently, this reduction in MYCN levels resulted in increased expression of total PKA C-α and CREB in NLF/TrkC cells (*p* = 0.0481 and *p* = 0.0293, respectively) suggesting an inverse relationship between these proteins (Fig. 4A).

Phenotypic analysis of siMYCN-NLF/TrkC cells stimulated with NT-3 showed an increase in neuronal differentiation and no concomitant increase in cell number, as compared to siNEG-NLF/TrkC cells (Fig. 4B–D).

To corroborate these findings, we also studied TrkC signalling in the MYCN-amplified IMR-32 neuroblastoma cells with high endogenous TrkC expression. Upon NT-3 treatment of IMR-32 cells, TrkC was phosphorylated and, similarly to NLF/TrkC cells, they showed no significant change in phospho-PKA levels (Fig. 4G). NT-3 stimulation led to an increase in cell number and no marked change in neuronal differentiation (Fig. 4E, F, H-J). Conversely, exogenous activation of the PKA pathway with db-cAMP reduced cell number and promoted neuronal differentiation in these cells (Fig. 4E, F, H–J). We further assessed differentiation by analysing the expression of tyrosine hydroxylase (TH), a marker of neuronal differentiation. Similarly to retinoic acid (RA), which is a clinically used differentiation agent for neuroblastoma, combined treatment with NT-3 and db-cAMP significantly increased the expression of TH after 5 days in IMR-32 cells (Fig. 4H, I). In IMR-32 cells, MYCN knockdown followed by NT-3 stimulation did not result in an increase in cell number compared with siNEG-IMR-32 cells (Fig. 4K). These results confirm that MYCN status alters NT-3/TrkC signalling and influences cell fate decisions of neuroblastoma cells.

Furthermore, to evaluate the in vivo efficacy of db-cAMP, fluorescently-labelled IMR-32 cells were transplanted into the perivitelline space of 2-day-old zebrafish embryos. Tumour progression was measured at 72 h post-injection, with comparisons made between xenografts treated with db-cAMP and untreated controls. Treatment with db-cAMP led to a significant regression of tumour mass in the xenotransplanted fish, as assessed by relative fluorescence intensity (Fig. 4L), further highlighting its therapeutic potential against MYCN-amplified neuroblastomas.

**Fig. 5:**
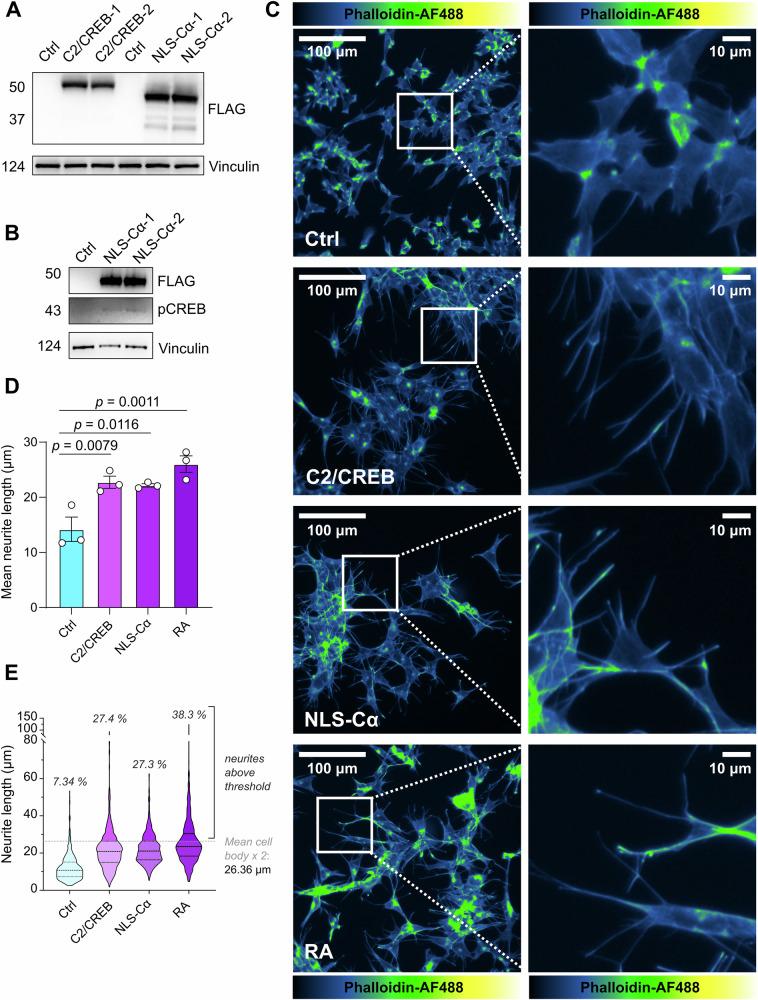
Overexpression of a constitutively active CREB mutant (C2/CREB) or the nuclear targeted catalytic subunit of PKA (NLS-Cα) in MYCN-amplified IMR-32 cells leads to differentiation. **A** Western blot analysis of IMR-32 cells transfected with an expression vector encoding C2/CREB or NLS-Cα (1 μg DNA). Western blots were probed with an antibody against the FLAG-tag. **B** Western blot analysis of p-CREB in IMR-32 cells 48 h following transfection with NLS-Cα (1 μg DNA). Vinculin was used as loading control. **C**–**E** Neuronal differentiation was assessed by phalloidin-Alexa Fluor 488 staining of IMR-32 cells transfected with C2/CREB or NLS-Cα for 7 days. 10 μM retinoic acid (RA) was used as a positive control to induce differentiation. (Mean ± SEM; *n* = 3; ANOVA with multiple comparisons to control).

To validate the role of PKA/CREB signalling in driving differentiation, we overexpressed a FLAG-tagged, constitutively active PKA (NLS-Cα) or CREB mutant (C2/CREB) in IMR-32 cells [44–46] (Fig. 5A, B). Morphological analysis revealed increased neurite length and increased percentage of differentiating cells upon overexpression of C2/CREB or NLS-Cα in IMR-32 cells (Fig. 5C–E). Taken together, these results indicate that in MYCN-amplified cells, reactivation of the PKA/CREB pathway promotes differentiation.

### Neuroblastoma patients with MYCN amplification show decreased expression of PKA pathway-related genes

**Fig. 6:**
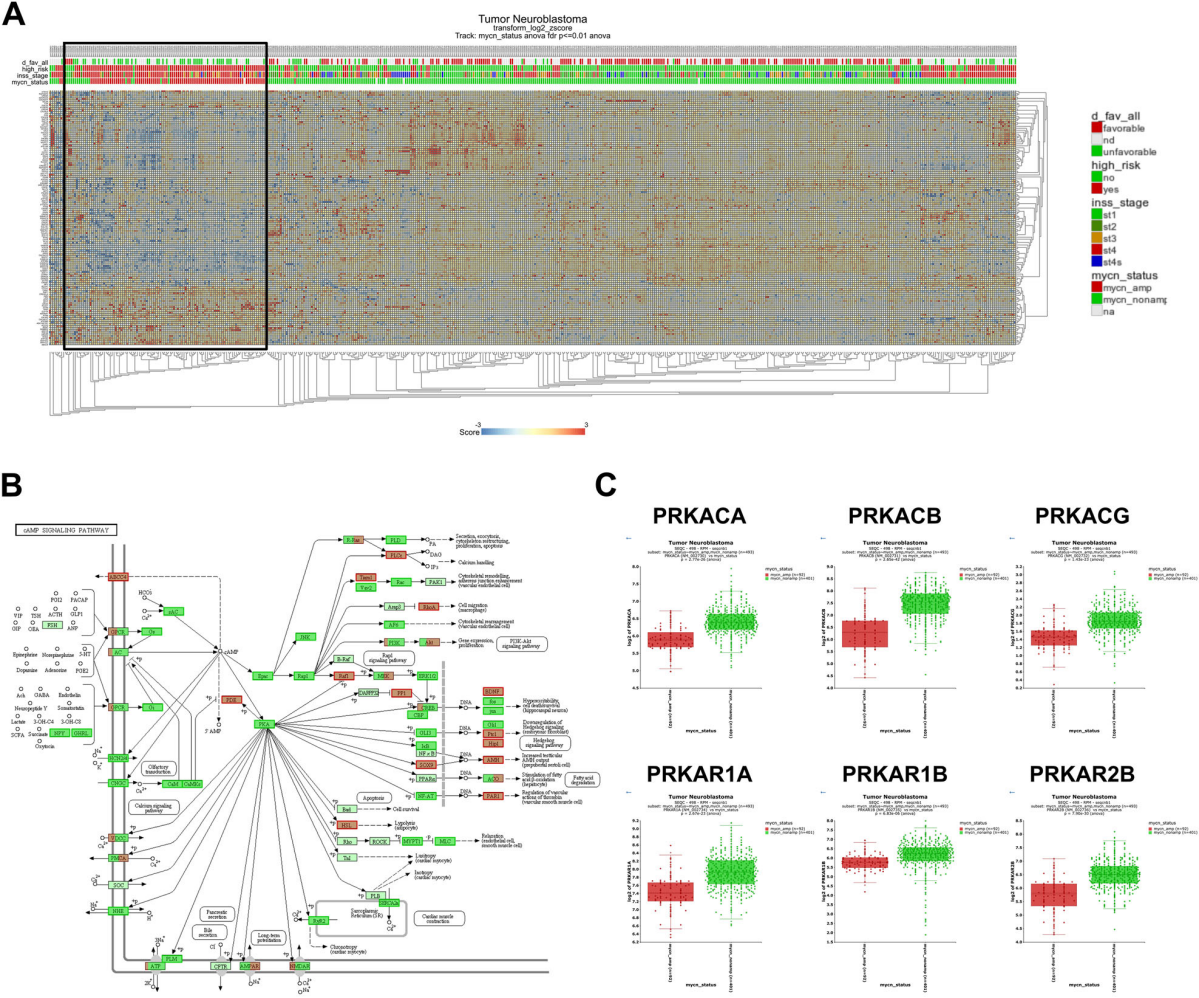
Genes related to the cAMP/PKA/CREB pathway are suppressed in MYCN-amplified patients. **A** Heat map representing the relative expression (mRNA) of cAMP/PKA/CREB pathway related genes within a cohort of 498 neuroblastoma patients (http://r2.amc.nl; SEQC-498-RPM-seqcnb1 dataset). Each column corresponds to one patient. The cluster of MYCN-amplified patients are highlighted by a square. **B** mRNA expression of components of the cAMP/PKA/CREB pathway (KEGG: hsa04024) in the neuroblastoma patient cohort. Green colour indicates significantly higher expression of genes in the MYCN non-amplified patients, and red colour indicates significantly higher expression of genes in the MYCN-amplified patients. (Light mint green indicates no significant difference between MYCN-amplified and non-amplified patients). **C** mRNA expression of PKA related genes in MYCN-amplified (red) vs MYCN non-amplified (green) neuroblastoma patients. **A**–**C** Were created using the R2: Genomics Analysis and Visualization Platform (http://r2.amc.nl).

To further examine the connection between MYCN status in neuroblastoma and cAMP/PKA/CREB pathway activity, we analysed a publicly available patient dataset comprising of 498 neuroblastoma patients (http://r2.amc.nl; SEQC-498-RPM-seqcnb1 neuroblastoma dataset). This dataset includes clinical annotations of MYCN amplification status, INSS staging, high-risk disease, age at diagnosis, and disease free survival, among others [47]. Of the 498 patients, 92 have MYCN amplification and 401 are MYCN non-amplified (5 not defined). Gene set analysis of the cAMP/PKA/CREB signalling pathway (hsa04024) showed that MYCN-amplified patients and the high-risk group cluster separately to MYCN non-amplified patients and have lower expression of genes involved in this pathway (Fig. 6A). Mapping of gene expression data onto the cAMP signalling pathway (KEGG: hsa04024) revealed that most proteins involved in this pathway are more highly expressed in MYCN non-amplified patients (Fig. 6B). Furthermore, 6 out of 7 key subunits of PKA, i.e., PKA C-α (gene: PRKACA), PKA C-β (gene: PRKACB), PKA C-γ (gene: PRKACG), PKA RI-α (gene: PRKAR1A), PKA RI-β (gene: PRKAR1B), PKA RII-β (gene: PRKAR2B), also display significantly lower gene expression in MYCN-amplified patients (Fig. 6C). There is no significant difference in the expression of the subunit PKA RII-α (gene: PRKAR2A). These findings suggest an interplay between MYCN amplification status and cAMP/PKA/CREB pathway activity in neuroblastoma.

To investigate if the PKA pathway related genes are transcriptional targets of MYCN, the CHEA Transcription Factor Targets dataset was queried through the Harmonizome platform [48]. PRKACA appears to be a MYCN target gene as well as other components of the PKA/CREB pathway including PRKAR2A, PRKAB1, CREB3L4, CREB5, CREBL2, AKAP1, AKAP12, AKAP8L, and AKAP9 (Figure S2A), suggesting that MYCN suppresses the transcription of PKA. In the MYCN-regulatable, MYCN-amplified cell line IMR-5/75 shMYCN, PRKACA expression increased following downregulation of MYCN (Figure S2B). Moreover, bioinformatic analysis of ChIP-Seq data (GSE184057 [49]) obtained from IMR-32 cells with siRNA-mediated knockdown of MYCN confirms that MYCN differentially binds to the PRKACA promoter region dependent on MYCN levels (Figure S2C). These findings further support that MYCN transcriptionally suppresses PKA.

### microRNA-221 positively correlates with MYCN expression in neuroblastoma and its downregulation increases CREB expression and neuronal differentiation

One mechanism by which MYCN rewires the cellular network is through the regulation of non-coding RNAs, including long non-coding RNAs, circular RNAs, and microRNAs [50–52]. Numerous studies have shown that the expression of microRNAs varies depending on MYCN levels in neuroblastoma [42, 52–54]. Of particular interest, the microRNA miR-221 has been previously demonstrated to be highly expressed in neuroblastoma tumours that overexpress MYCN. Studies have shown its induction by MYCN in neuroblastoma, and that it positively correlates with poor patient survival [54, 55]. Additionally, its suppression is considered important for cAMP/PKA/CREB pathway activation and neuronal differentiation in glioma cells [56]. Based on this, we investigated miR-221 as a potential mechanism of regulation.

**Fig. 7:**
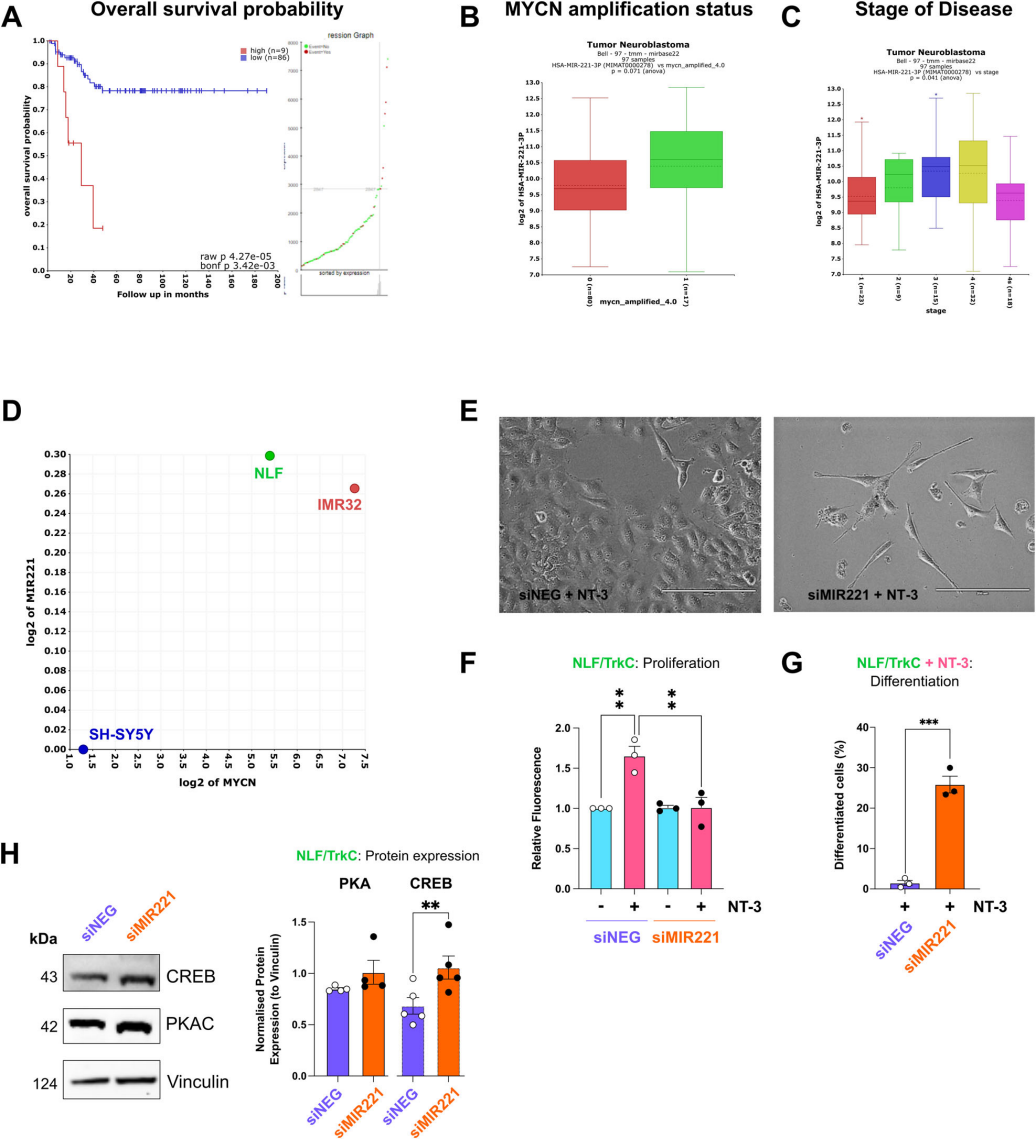
microRNA-221 positively correlates with MYCN expression in neuroblastoma and its downregulation increases CREB expression and neuronal differentiation. **A** Kaplan-Meier curves representing the overall survival of patients with high (red) vs low (blue) miR-221 expression. (KaplanScan method was employed to differentiate high vs low expression). **B** Expression of miR-221 in MYCN non-amplified (red) vs MYCN-amplified (pink) neuroblastoma patients (Bell dataset). **C** Expression of miR-221 across different stages of neuroblastoma. **D** Correlation of MYCN levels with miR-221 in neuroblastoma cell lines used in this study (Maris-41-fpkm-rsg001). **A**–**D** Were created using the R2: Genomics Analysis and Visualization Platform (http://r2.amc.nl). **E** Representative images of phenotypic observation by light microscopy after 5 days of treatment with NT-3 (100 ng/ml) in NLF/TrkC cells with siRNA-mediated knockdown of MIR221 (50 nM) or non-targeting control. Magnification: X20; scalebar: 200 µm. **F** Quantification of mean fluorescence as a measure of cell proliferation by CyQuant in NLF/TrkC cells following siRNA-mediated knockdown of MIR221 (50 nM) or non-targeting control (siNEG) following treatment with NT-3 (100 ng/ml) for 72 h. **G** Quantification of neuronal differentiation with NeuronJ in NLF/TrkC cells with siRNA-mediated knockdown of MIR221 (50 nM) or non-targeting control (siNEG) following treatment with NT-3 (100 ng/ml) for 5 days. **H** Western blot analyses of PKA C and CREB protein expression following siRNA-mediated knockdown of MIR221 (50 nM) or non-targeting control (siNEG) for 24 h in NLF/TrkC cells. Data presented as protein expression compared to loading control (vinculin). Graphs show data as mean ± SEM; *n* = 3 (*p* < 0.05 = *, *p* < 0.001 = **).

Further bioinformatic analysis of miR-221 in patient data using the Bell dataset [53] highlighted significant differences in miR-221 expression across subtypes of neuroblastoma patients (Fig. 7A-C). Elevated miR-221 expression was associated with unfavourable characteristics, including MYCN amplification, decreased overall survival, and stage 4 disease.

Additionally, utilising publicly available transcriptomic data of neuroblastoma cell lines (Maris-41-fpkm-rsg001) [57], we observed a strong positive correlation between the expression levels of MYCN and MIR221 in SH-SY5Y, NLF and IMR-32 neuroblastoma cells used in this study (*R*-value = 0.916) (Fig. 7D). Knockdown of MIR221 in NLF/TrkC cells treated with NT-3 induced a more favourable phenotype by promotion of neuronal differentiation and mitigation of cell proliferation (Fig. 7E–G). Furthermore, MIR221 knockdown increased total CREB expression, while PKA levels were not significantly different (Fig. 7H). Collectively, these findings suggest that high levels of miR-221 in MYCN-amplified neuroblastoma cells suppress the PKA/CREB pathway, consequently inhibiting TrkC-mediated neuronal differentiation.

## Discussion

Using quantitative mass spectrometry-based phosphoproteomics, we defined the temporal landscape of NT-3/TrkC signalling in neuroblastoma across distinct MYCN contexts. Analysis of TrkC-overexpressing neuroblastoma cell lines revealed a clear MYCN-dependent rewiring of downstream signalling and cellular phenotype. NT-3 stimulation promoted neuronal differentiation in MYCN non-amplified cells but induced proliferation in MYCN-overexpressing or MYCN-amplified cells. This phenotype was reversible upon siRNA-mediated MYCN knockdown, establishing MYCN as a key determinant of TrkC functional output. At a global level, MYCN also reshaped NT-3/TrkC signalling dynamics, as demonstrated by distinct PCA clustering of phosphoproteomic profiles according to MYCN status.

We identified differential activation of the cAMP/PKA/CREB pathway following NT-3 stimulation as a central MYCN-dependent signalling event governing cell fate decision. PKA signalling has previously been implicated in differentiation of neural stem cells, hippocampal progenitors and pluripotent stem cells [58–61]. Moreover, Sánchez et al. also recognised cAMP-mediated neurite formation in neuroblastoma cells. Their results demonstrated both PKA and PI3K activation to be essential for this phenotype [62]. Consistent with this, PI3K/AKT-related pathways were enriched in our kinase substrate enrichment analysis of SH-SY5Y/TrkC cells. However, PI3K/AKT activity did not differ significantly between MYCN non-amplified and MYCN-amplified TrkC-expressing cells following NT-3 stimulation, and neither pharmacological inhibition nor constitutive activation of AKT affected neuronal differentiation (Figure S3), indicating that PI3K/AKT signalling is not the primary driver of TrkC-mediated differentiation in this context.

MYCN knockdown in MYCN-amplified cells resulted in upregulation of PKA and CREB protein expression, suggesting that MYCN suppresses NT-3/TrkC-induced differentiation through inhibition of the PKA/CREB axis. Consistently, reactivation of PKA/CREB signalling promoted differentiation in MYCN-amplified cells. These findings were further supported by patient data, which showed reduced expression of PKA-related genes and overall suppression of PKA/CREB pathway components in MYCN-amplified neuroblastomas. The correlation between our in vitro findings and clinical data reinforces that our results accurately reflect the role of MYCN biology within the clinical context of neuroblastoma. These findings are also in agreement with other studies that highlighted CREB’s involvement in neuronal differentiation and suppressing neuroblastoma tumour growth [63, 64].

Our data further suggest that MYCN suppresses CREB expression, at least in part, via upregulation of miR-221, a microRNA associated with unfavourable patient outcomes in neuroblastoma [53–55]. In line with previous reports showing that miR-221 inhibition activates PKA/CREB signalling and induces differentiation in glioma cells [56], we propose that MYCN-driven miR-221 expression contributes to suppression of TrkC-mediated differentiation. Target prediction analysis (using the TargetScan database) identified NT-3 itself as a potential miR-221 target [65], raising the possibility that miR-221 may dampen TrkC signalling at multiple levels. Interestingly, while miR-221 directly suppresses CREB, PKA expression was unaffected by modulation of miR-221 levels. Instead, PKA expression responds to MYCN knockdown, suggesting that MYCN also regulates components of the PKA/CREB pathway at a transcriptional level (Figure S2).

Beyond transcriptional control, MYCN also influenced PKA signalling spatially through regulation of A-kinase anchoring proteins (AKAPs) that control the subcellular localisation of PKA. Of note, we identified AKAP12 as upregulated and more active in MYCN-high cells (Figure S4). Given its role in tethering PKA to the plasma membrane [66, 67], increased AKAP12 expression may restrict PKA nuclear translocation and subsequently limit CREB activation, providing an additional mechanism by which MYCN suppresses neuronal differentiation.

Calcium signalling may represent an additional mechanism through which MYCN regulates the cAMP/PKA pathway. Calcium influx is an upstream activator of cAMP/PKA signalling, and unfavourable neuroblastomas exhibit dysregulated calcium signalling and silencing of calcium-sensing genes, whereas these genes are highly expressed in differentiated, favourable tumours [68–70]. Notably, calreticulin (CALR) has independent prognostic value in neuroblastoma, with higher expression associated with favourable clinical features, including younger age at diagnosis, early-stage disease, differentiated histology, and absence of MYCN amplification. In our proteomics data, CALR is elevated in SH-SY5Y/TrkC cells compared with NBLS/TrkC and NLF/TrkC cells (Figure S5). These observations may suggest a mechanistic link between calcium signalling and MYCN-driven regulation of the PKA pathway, warranting further investigation into its role in neuroblastoma differentiation.

Beyond the specific mechanisms identified here, it is important to consider the broader transcriptional landscape governed by MYCN, which is known to reshape gene expression programs globally in neuroblastoma. As a transcription factor, MYCN regulates genes involved in signal transduction, metabolism, chromatin organisation, and microRNA biogenesis, any of which may indirectly modulate the cAMP/PKA/CREB axis. Thus, MYCN-mediated suppression of TrkC-driven differentiation is likely to reflect coordinated transcriptional and post-transcriptional regulation across multiple signalling nodes.

Importantly, our study also identifies a therapeutic vulnerability in MYCN-amplified neuroblastoma. Differentiation of neuroblasts to mature benign neuronal cells is a therapeutic goal in the treatment of high-risk neuroblastoma. Low stage tumours show a more differentiated phenotype, which has been proposed as a mechanism driving the spontaneous regression of stage 4S tumours [71]. Differentiation is currently achieved in the clinic during maintenance therapy by administration of all-trans retinoic acid to patients [72, 73]. Owing to its effectiveness being limited to minimal residual disease, much research efforts are ongoing to identify novel differentiation agents or agents to potentiate the effects of retinoic acid. Our data suggest that pharmacological activation of the cAMP/PKA/CREB pathway may represent an alternative or complementary differentiation strategy. Indeed, the cAMP analogue tocladesine (8-Cl-cAMP) has been previously used in phase I/II clinical trials in colorectal cancer, multiple myeloma, and plasma cell neoplasms (NCT00004902, NCT00021268), highlighting the translational feasibility of this approach. Further exploration is warranted to fully elucidate its therapeutic potential in neuroblastoma.

In conclusion, our work provides a comprehensive map of NT-3/TrkC signalling in neuroblastoma and illustrates how MYCN reprograms this pathway to dictate cell fate. We identify the cAMP/PKA/CREB axis as a critical mediator of TrkC-induced neuronal differentiation and propose that MYCN suppresses this pathway through both transcriptional regulation and miR-221 upregulation. Targeting this signalling axis may hold promise for differentiation-based therapies in MYCN-amplified neuroblastoma.

## Materials and methods

### Antibodies, ligands and inhibitors

We used the Phospho-TrkA (Tyr490)/TrkB (Tyr516) (C35G9) Rabbit mAb (#4619, Cell Signaling) to detect pTrkC as the key phosphorylation sites, including Tyr490 of TrkA, are conserved between the human TrkA, TrkB and TrkC receptors. Tyr490 of TrkA corresponds to Tyr516 in TrkB, and Tyr516 in TrkC. Other antibodies used include TrkC (C44H5) (#3376), Phospho-ERK (Thr^202^/Tyr^204^) (#4370), Phospho-Akt (Ser^473^) (#4060), Akt (#9272), Phospho-PKA C (Thr^197^) (#4781), PKA C-α (#4782), Vinculin (#4650), CREB (#9197), Phospho-CREB (Ser^133^) (#9191), GAP43 (#8945), Tyrosine hydroxylase (#2792), GSK-3β (#12456), and GAPDH (14C10) (#2118), from Cell Signalling Technology. V5-tag monoclonal antibody (#R960-25) and Phospho-PLCγ(Tyr^783^) (#44-696) were from Invitrogen; and the MYCN antibody (#sc-53993) was from Santa Cruz Biotechnology. Secondary horseradish peroxidase–conjugated antibodies against rabbit (#7074) or mouse (#7076) immunoglobulin G (IgG) were from Cell Signaling Technology.

Recombinant Human NT-3 (#450-03) was obtained from Peprotech. The PKA inhibitor, H 89 2HCl (#S1582) and the PKA activator, dibutyryl-cAMP (#S7858) were purchased from Selleck Chemicals. Retinoic acid (RA; #R2625) was from Sigma-Aldrich.

### Cell lines

The human neuroblastoma cell lines SH-SY5Y, NBLS and IMR-32 (Table 1) were generous gifts from Frank Westermann (Deutsches Krebsforschungszentrum (DKFZ), Heidelberg, DE) and the NLF cell line was obtained from Kerafast (ECP008). Cell lines were cultured in RPMI 1640 (Gibco) supplemented with 10% (v/v) foetal bovine serum (Gibco), 2 mM L-glutamine (Gibco), and penicillin (100 U/ml) and streptomycin (100 μg/ml) (Gibco). The media for cell lines expressing the pLX302/NTRK3 plasmid were additionally supplemented with 1 μg/ml puromycin (Sigma-Aldrich) to maintain plasmid expression. All cell lines were routinely tested for Mycoplasma.

### Generation of pLX302/NTRK3 cell lines

Cells grown on 100 mm dishes were transfected with 5 μg of DNA using jetPRIME transfection reagent (Polyplus) according to the manufacturer’s instructions. After 24 h, the media was changed to RPMI 1640 full media containing puromycin (1 μg/ml) to allow antibiotic selection of positively transfected cells.

### Cell stimulation and lysis

Cells were serum starved with media containing 0.1% FBS for 6 h prior to treatment. After starvation, cells were treated with NT-3 (100 ng/ml) ±  inhibitor for time-course stimulation. Cells were lysed in lysis buffer (1% Triton X-100, 20 mM Tris-HCl pH 7.5, 150 mM NaCl, 1 mM MgCl_2_), supplemented with protease inhibitor Complete Mini (Roche) and phosphatase inhibitor PhosStop (Roche). Lysates were cleared by centrifugation (14000 rpm, 10 min, 4 °C) and the supernatants were stored at −20 °C until analysis.

### Western blotting

SDS-polyacrylamide gel electrophoresis (SDS-PAGE) and Western blotting were performed using the Bolt Mini Gel Tank and Bolt Bis-Tris Precast 10% gels (Invitrogen). Gels were transferred onto PVDF membranes using the XCell II Blot Module system (Invitrogen). Membranes were blocked in 5% non-fat dried milk (Sigma-Aldrich) for 1 h at room temperature prior to overnight incubation at 4 °C with primary antibody diluted in bovine serum albumin (BSA) (1:1000). Membranes were washed in TBS-Tween (3 ×5 min) then incubated with the corresponding secondary antibodies diluted in 5% milk (1:5000) for 1 h at room temperature. Blots were washed and then developed using the iBright imaging system (ThermoFisher Scientific) with ECL or SuperSignal West Femto Chemiluminescent Substrate (ThermoFisher Scientific). Quantification of blots was achieved using ImageJ software v1.44p (http://imagej.nih.gov/ij).

### Cell proliferation

Cells were seeded in technical triplicates at 5000 cells/well in 96-well plate format. After 4 h, cells were treated with NT-3 (100 ng/ml) ±  inhibitors. At 72 h post treatment, cell proliferation was measured using the CyQUANT™ Assay (ThermoFisher Scientific) according to the manufacturer’s instructions. Fluorescence was measured using the SpetraMax M3 plate reader.

### Neurite outgrowth analysis

Cells (5 × 10^3^/ml) were treated with NT-3 (100 ng/ml) ±  inhibitor in 6-well plate format. Media and treatment were refreshed every 2 days. Using light microscopy, images were taken at day 5 using EVOS FL Imaging System microscope (20× magnification). Neurite elongation and cell number was measured on three images from each experimental condition using NeuronJ Software [69] and cellpose3 package in Python [70], respectively. NBLS/Trk C cells were counted manually due to segmentation issues in cellpose3.

For phalloidin staining, cells were plated in phenol red-free complete media (Gibco). After 7 days, cells were fixed, permeabilized and Alexa Fluor 488 Phalloidin (Invitrogen) labelling was performed according to the manufacturer’s instructions. Images were taken with an EVOS Digital Inverted Fluorescence Microscope using the FITC (green) filter. Cells with outgrowths at least twice the length of their cell body diameter were considered differentiated.

### Luciferase reporter assay

The CRE/CREB Reporter Kit (cAMP/PKA Signalling Pathway) (Generon) and BPS Two-Step Luciferase (Firefly & Renilla) Assay System was used according to manufacturer’s instructions and measured using the SpetraMax M3 plate reader.

### Plasmids and siRNA-mediated knockdown

The expression vectors pFUW-FLAG-NLS-Cα and pFUW-FLAG-C2/CREB were generous gifts from Prof Gerald Thiel (Saarland University, Germany). The expression vector pFUW-FLAG-NLS-Cα encodes the catalytic subunit of protein kinase A together with a nuclear localization signal derived from the SV40 large T antigen [44, 46]. The pFUW-FLAG-C2/CREB expression vector encodes the constitutively active transcriptional activation domain of CREB2 and the bZIP domain of CREB [45].

The MYCN (#L-003913-01-0005), MIR221 Silencer^®^ Select (#4390771) and negative control (D-001810-10-05) ON-TARGETplus SMARTpool siRNAs were purchased from ThermoFisher Scientific.

Transfections were performed using jetPRIME reagent (Polyplus) according to manufacturer’s instructions.

### Liquid chromatography tandem mass spectrometry (LC-MS/MS)

Samples for mass spectrometry were prepared as described in Maher et al. [74]. Samples were run on a Bruker timsTof Pro mass spectrometer connected to a Evosep One liquid chromatography system as described previously [75].

The mass spectrometer raw files were searched against the Homo sapiens subset of the Uniprot Swissprot database (reviewed) using the search engine FragPipe (Version 18) [75].

### Bioinformatics

The mass spectrometry proteomics data have been deposited to the ProteomeXchange Consortium via the PRIDE [76] partner repository with the dataset identifier PXD054441 [77]. Data analysis was performed in R (Version 4.1.2). LFQ intensities were log2-transformed. Proteins/phosphosites with more that 80% missing values in all conditions were filtered out. Missing values were imputed using the group mean imputation with normal distribution correction and tail-based imputation approach. Normalized and imputed data are available at figshare [78]. Analysis of differently expressed phosphosites and proteins was performed using the limma package in R/Bioconductor [79] with adjusted *p* value < 0.05 and absolute fold change > 1.5 as the cutoffs for a phosphosite to be considered significantly different compared to the zero timepoint of each experimental condition. Kinase substrate enrichment analysis was performed using the KSEAapp R package [80, 81] with default parameters.

For transcription regulation analysis, the CHEA database in Harmonizome 3.0 was used to identify PKA pathway-related genes transcriptionally regulated by MYCN [48]. ChIP-seq data from IMR-32 cells treated with siMYCN or control siRNA [49] were analysed using R2 Genomics Analysis and Visualization Platform (http://r2.amc.nl) to visualise MYCN binding at the PRKACA locus.

### Zebrafish xenograft experiments

All zebrafish experiments were conducted in accordance with ethical guidelines and were approved by the University College Dublin Animal Research Ethics Committee and the Health Products Regulatory Authority (AE18982/P253). Adult zebrafish (*Danio rerio*) were housed at 28.5 °C in a recirculating water system, under a 14-h light/10-h dark cycle. At 2 days post-fertilization (dpf), zebrafish embryos were manually dechorionated using fine forceps. The dechorionated embryos were transferred to a pre-warmed 2% agarose gel and anaesthetised with Tricaine (0.05 mg/ml). IMR-32 neuroblastoma cells were fluorescently labelled with DiI (Invitrogen) lipophilic membrane stain, and approximately 100–500 cells were microinjected into the perivitelline space of each embryo. Following microinjection, the embryos were screened for successful injections, imaged, and randomly assigned to an individual well of a 24-well plate containing 1 mL of 0.003% 1-phenyl 2-thiourea (PTU) solution ± db-cAMP (1 mM). Embryos were incubated at 35 °C for a treatment period of 72 h. Following this, tumour progression was assessed using fluorescent microscopy (EVOS FL Imaging System).

### Patient data

Analysis of neuroblastoma patient gene expression data was performed using the R2 Genomics Analysis and Visualization Platform (http://r2.amc.nl). The SEQC-498-RPM-seqcnb1, Kocak - 649 - custom - ag44kcwolf data set and TARGET - 161 – fpkm bulk RNA-Seq datasets were used. Gene set analysis of KEGG pathways (hsa04024) was performed (*p* value cut-off < 0.05). Analysis of individual genes was done by comparing the log2 gene expression separated by MYCN-status (MYCN-amplified vs MYCN non-amplified, NA samples were excluded). Expression was considered significantly different if *p* value < 0.05.

### Statistical analysis

All experiments were performed in three biological replicates. Statistical analyses for cell-based assays and Western blots were conducted using GraphPad Prism (version 8.0.2). Data are expressed as mean ± SEM. Student *t* test was used for comparison between two groups or one-way Anova for more than two groups. *P* value < 0.05 were considered statistically significant; *p value < 0.05, **p value < 0.01, ***p value < 0.001.

## Supplementary information

Supplemental Figures

Supplemental Western blots

## Data Availability

The mass spectrometry proteomics data have been deposited to the ProteomeXchange Consortium via the PRIDE partner repository with the dataset identifier PXD054441 [77]. Normalised and imputed data are available at figshare [78].
