# Supplementary material for: MYCN inhibits TrkC-mediated differentiation in neuroblastoma cells via disruption of the PKA signalling pathway
Source: Cell Death Discov. 2026 Mar 25;12:176. doi: 10.1038/s41420-026-03024-y (PMC13039803; doi:10.1038/s41420-026-03024-y)
Supplement: Supplementary file 1 — Supplemental Figures [file 41420_2026_3024_MOESM1_ESM.pdf]

Manuscript Number: **CDDISCOVERY-25-1371R**

Title: **MYCN inhibits TrkC-mediated differentiation in neuroblastoma cells via disruption of the PKA signalling pathway**

Authors: Stephanie Maher, Andrew Roe, Kieran Wynne, Vadim Zhernovkov, Melinda Halasz

Corresponding Author: melinda.halasz@ucd.ie

This PDF file includes:

Supplementary Figures 1-5

Supplementary Figure 1

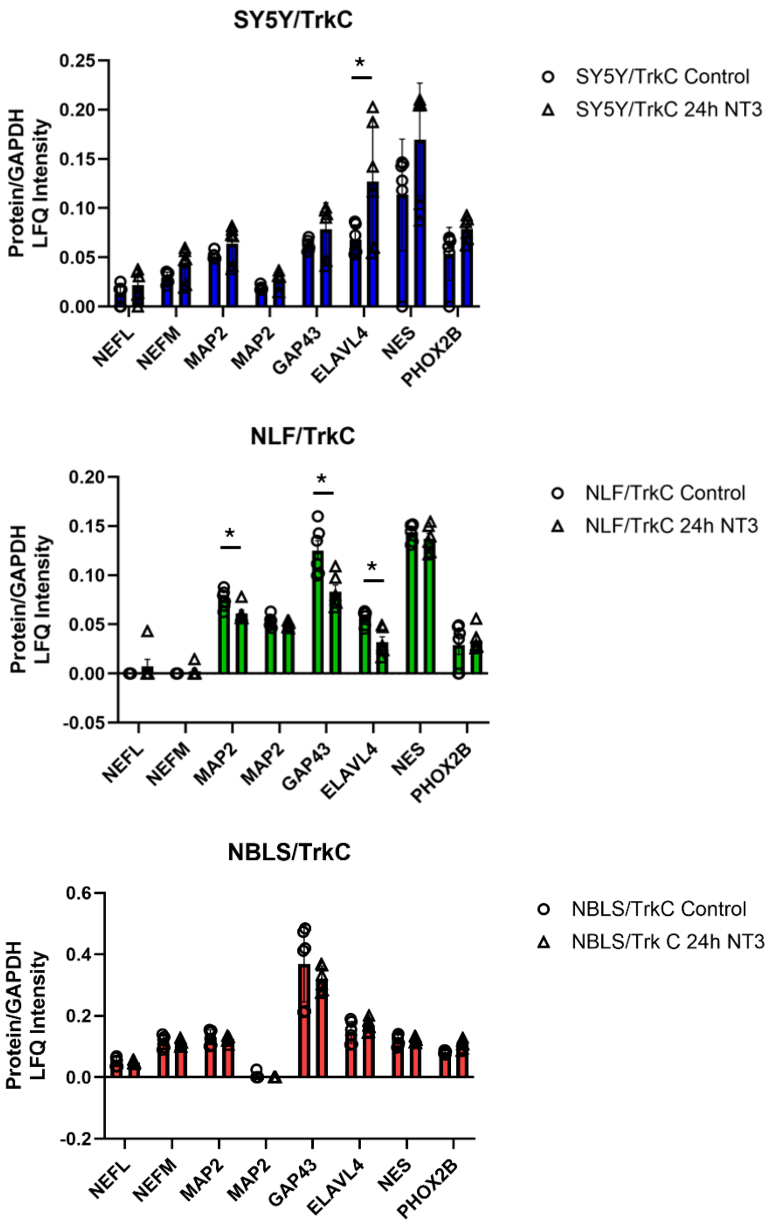

Figure S1. Neuronal differentiation markers increase at the protein level upon NT-3 treatment in SY5Y/TrkC cells while decrease in NLF/TrkC cells. Protein expression of neurofilaments (NEFL and NEFM), MAP2 (isoforms 3 and 4), GAP43, ELAVL4, NES and PHOX2B in SY5Y/TrkC, NLF/TrkC and NBLS/TrkC cells treated with NT-3 for 24 hours. Protein expression is displayed as the mean ± SEM of label-free quantification (LFQ) intensities normalized to GAPDH LFQ (LC-MS/MS). Each condition includes 3 biological and 2 technical replicates.

Supplementary Figure 2

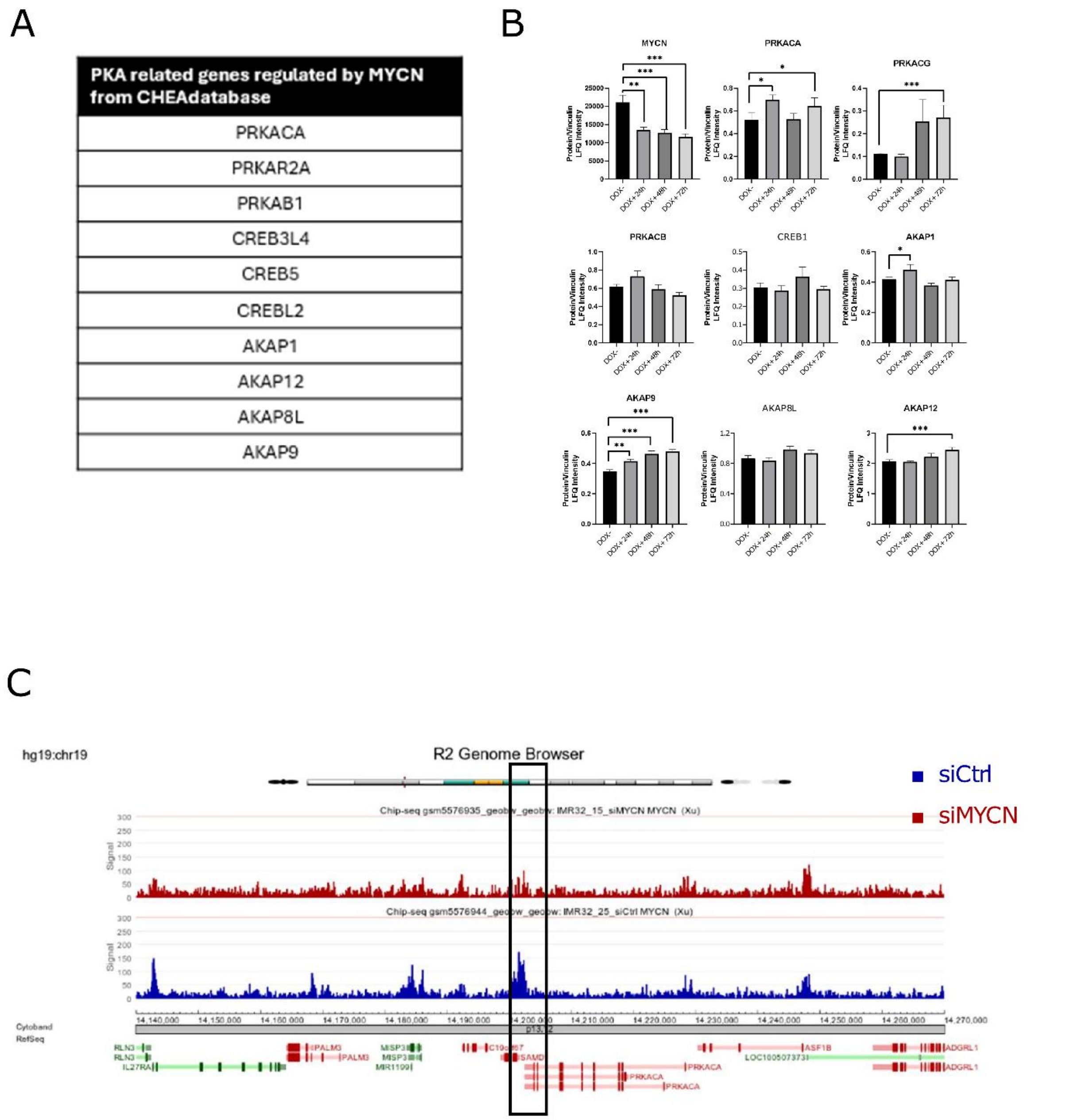

Figure S2. PKA and related genes are MYCN transcriptional targets. A) A list of PKA pathway related genes that are transcriptional targets of MYCN from the CHEA database (34). B) Protein expression of PKA-related proteins in IMR-5/75 shMYCN cells where MYCN is down-regulatable by doxycycline (DOX). Cells were treated with DOX for 24, 48, and 72 hours to induce MYCN downregulation and subjected to LC-MS/MS for proteome quantification. Protein expression is displayed as the mean ± SEM of label-free quantification (LFQ)

intensities normalized to vinculin LFQ. Each condition includes 3 biological and 2 technical replicates. C) ChIP-seq signals across the PRKACA gene region for experimental conditions IMR-32 cells treated with siMYCN (red) versus siCtrl (blue) from Xu et al. (35). The highlighted region (black box) indicates locus of interest with significant differences in signal intensity between the MYCN knockdown (siMYCN) and control (siCtrl) samples. Data analysis conducted using the R2: Genomics Analysis and Visualization Platform (http://r2.amc.nl).

Supplementary Figure 3

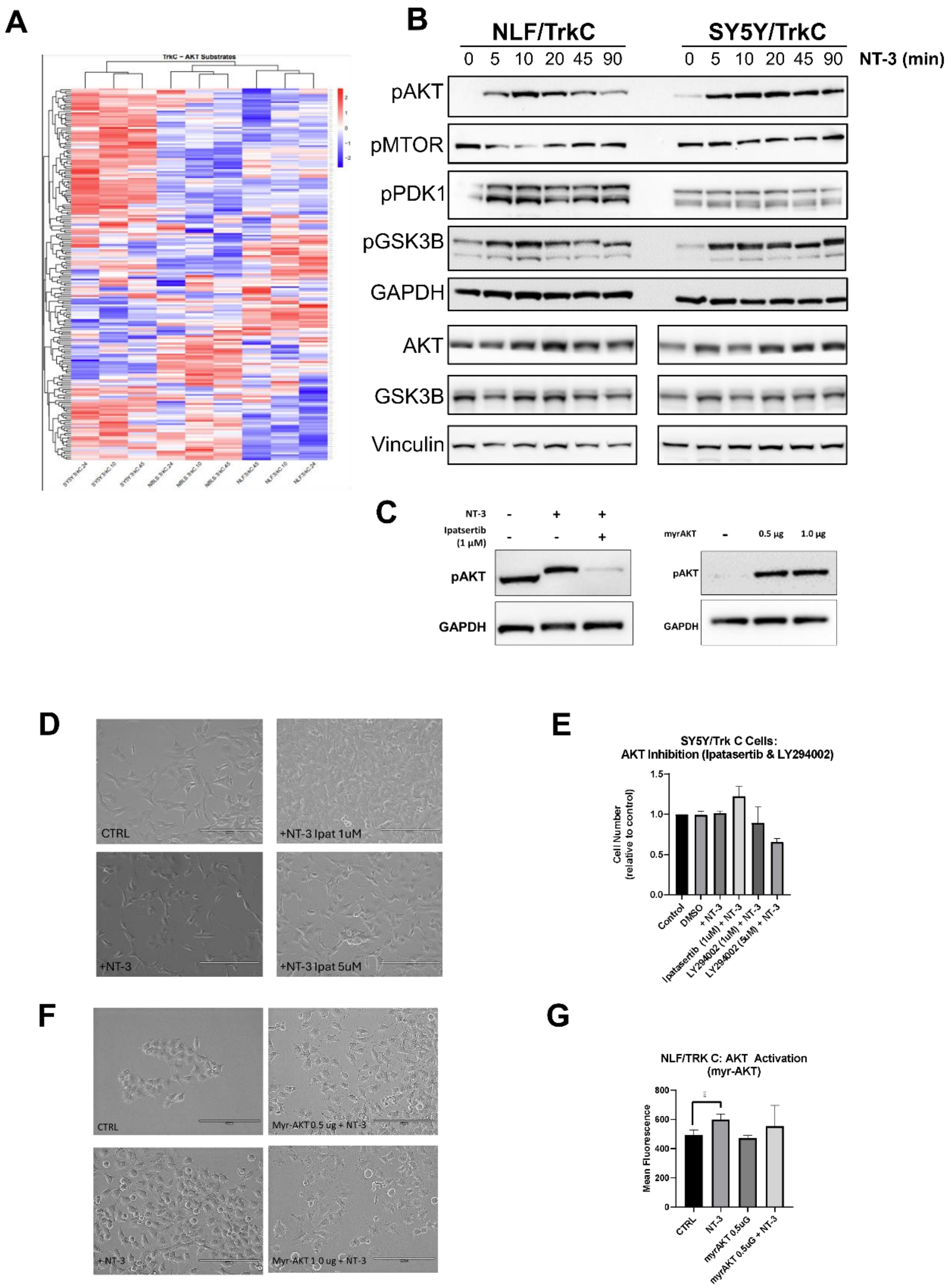

Figure S3. Investigation of AKT signalling & cell fate decisions in TrkC cells. A) Differences in AKT substrate phospho-activity following NT-3 stimulation in SH-SY5Y/TrkC cells, NBLS/TrkC and NLF/TrkC cells. **B)** Western blot analysis of pAKT, pMTOR, pPDK1,

pGSK3β, AKT, and GSK3β following stimulation of SH-SY5Y/TrkC cells and NLF/TrkC cells with NT-3 for 0-5-10-20-45-90 min. GAPDH, vinculin: loading controls. **C)** Confirmation of AKT inhibition with Ipatsertib (1uM) and activation of AKT with myristoylated-AKT plasmid (0.5 μg & 1.0 μg). **D)** Phenotypic observation following Ipatsertib (1 μM) or **F)** myr-AKT treatment. **E)** Measure of cell proliferation using CyQuant assay following 72 h of treatment as in D) in SH-SY5Y/Trk cells. **G)** Measure of cell proliferation using CyQuant assay following 72 h of treatment as in F) in NLF/TrkC cells. Data was quantified as mean fluorescence as a measure of relative cell number. Data is presented as mean ± SEM, n=3.

Supplementary Figure 4

A

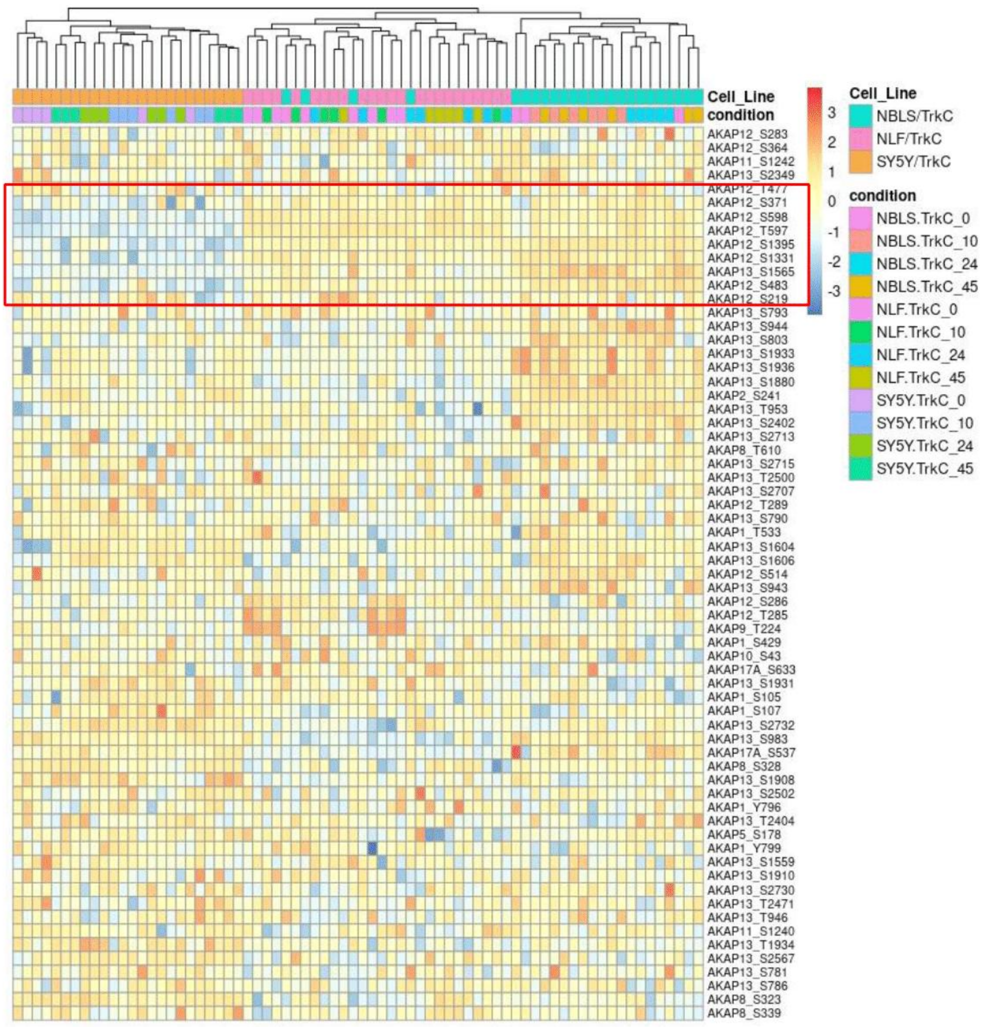

B

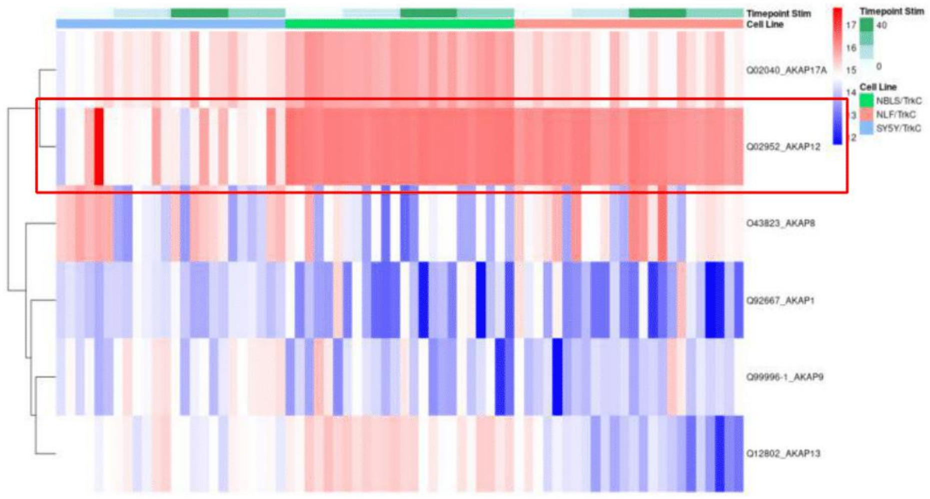

Figure S4. AKAP12 is differently expressed and activated in MCN-low vs MYCN-high cells. A) AKAP phosphosite activity in SH-SY5Y/TrkC, NBLS/TrkC and NLF/TrkC cells stimulated with NT-3 (100 ng/ml) for 10 min, 45 min, 24 hours. Each condition includes 3 biological and 2 technical replicates. **B)** Expression of AKAP proteins in SH-SY5Y/TrkC, NBLS/TrkC and NLF/TrkC cells in total proteomics data.

Supplementary Figure 5

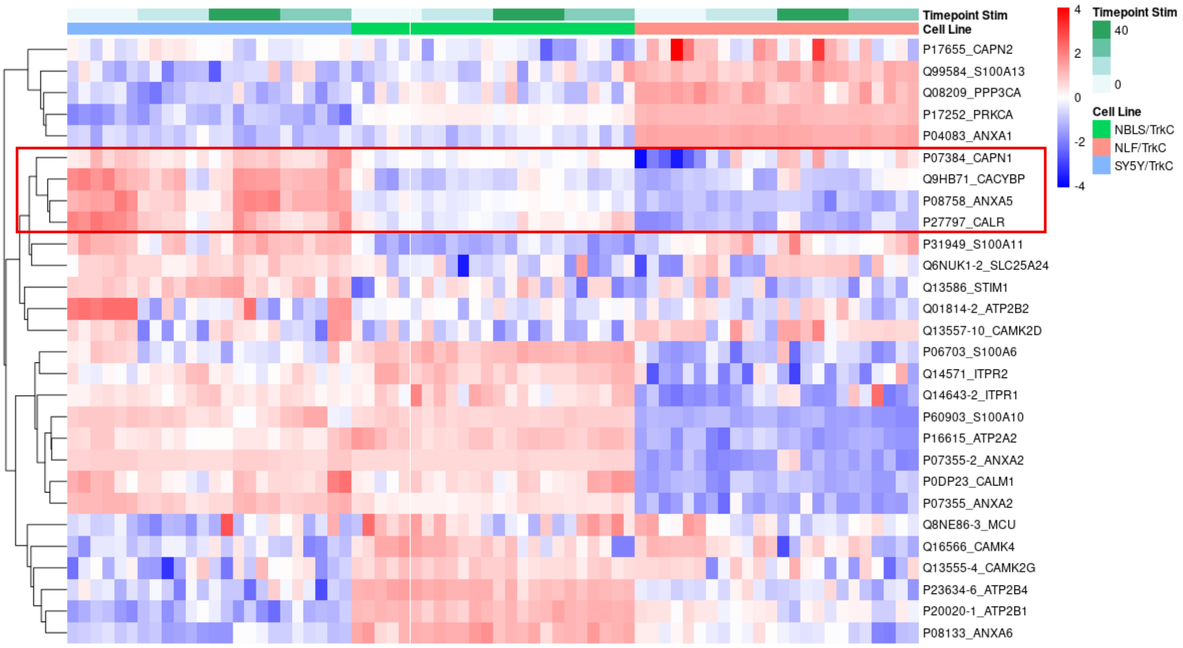

Figure S5. Calreticulin (CALR) and calcyclin-binding protein (CACYBP) are differentially expressed in MCN-low vs MYCN-high cells. Expression of calcium-related proteins in SH-SY5Y/TrkC, NBLS/TrkC and NLF/TrkC cells in total proteomics data. Cells were stimulated with NT-3 (100 ng/ml) for 10 min, 45 min, 24 hours. Each condition includes 3 biological and 2 technical replicates.
