# Supplementary material for: MYCN inhibits TrkC-mediated differentiation in neuroblastoma cells via disruption of the PKA signalling pathway
Source: Cell Death Discov. 2026 Mar 25;12:176. doi: 10.1038/s41420-026-03024-y (PMC13039803; doi:10.1038/s41420-026-03024-y)

Original blots from Figure 1

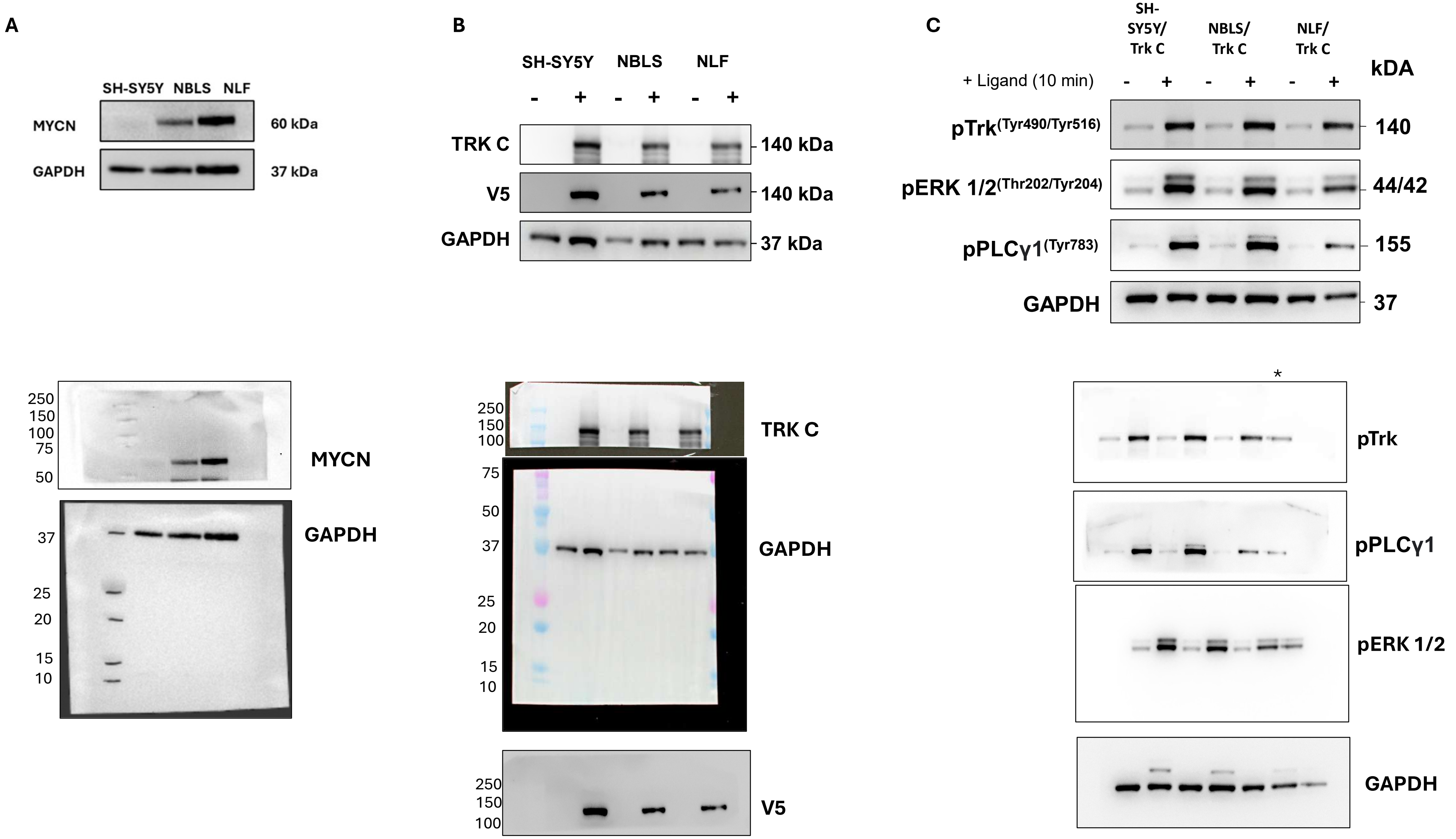

*Last lane (sample 6) had leak into the next lane (lane 7)

Original blots from Figure 3

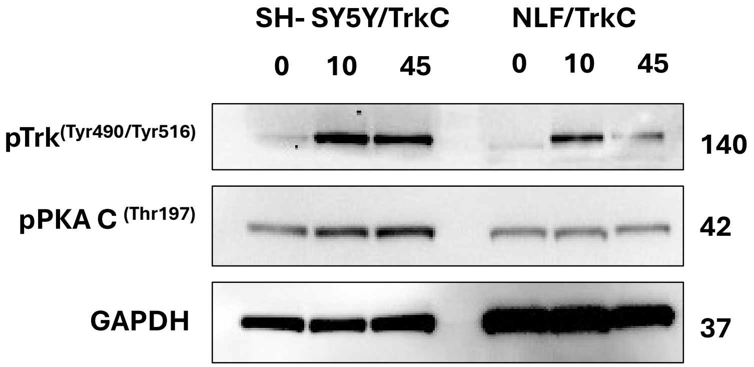

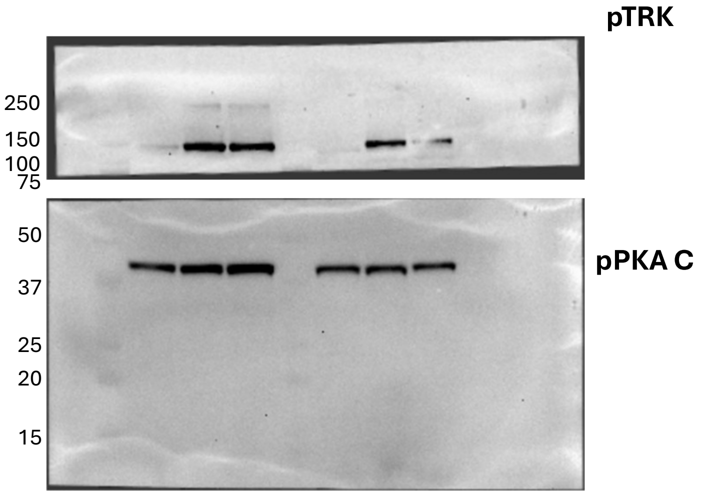

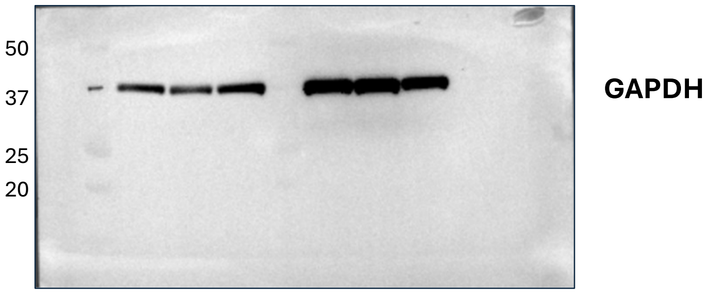

Original blots from Figure 4

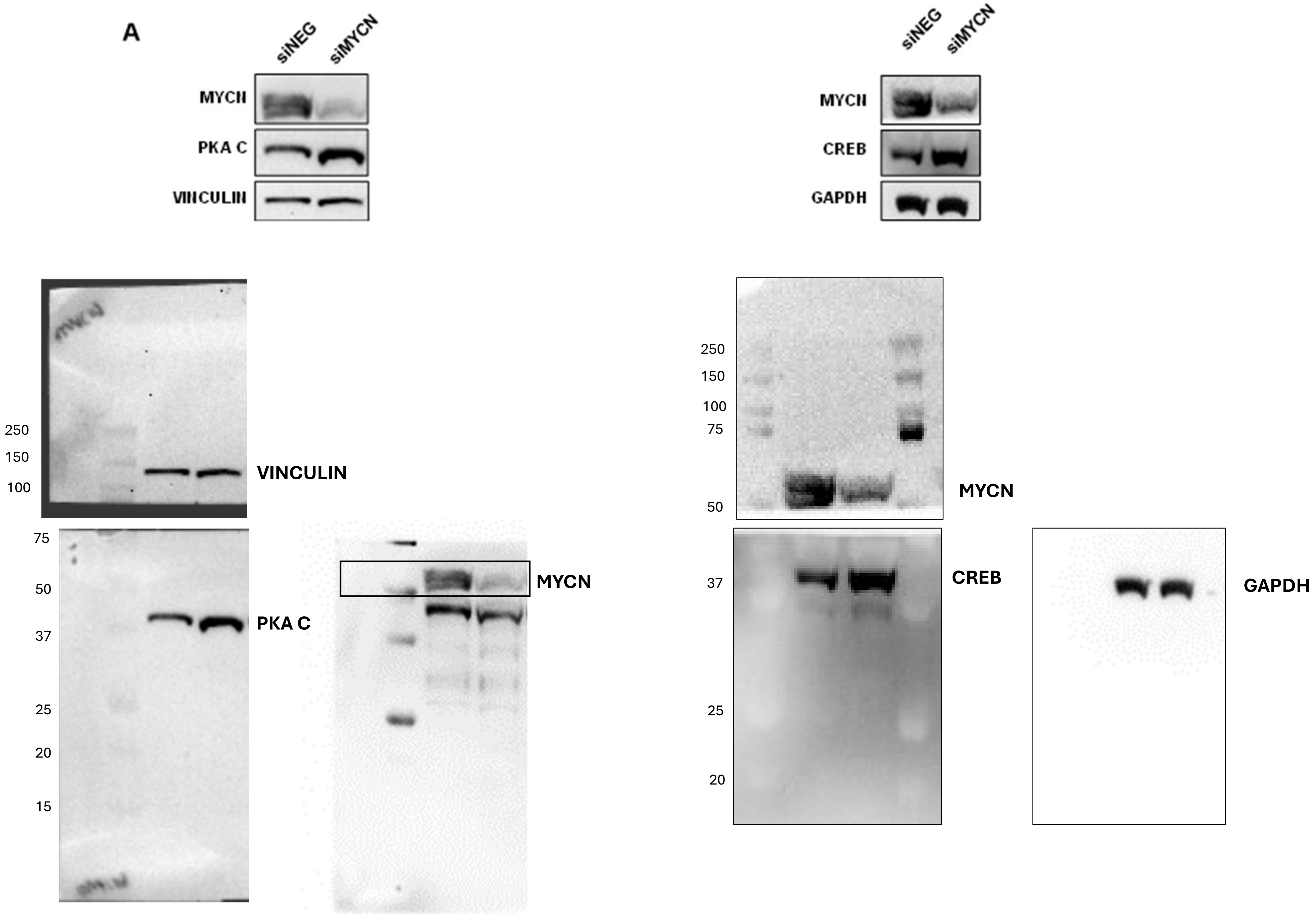

Original blots from Figure 4

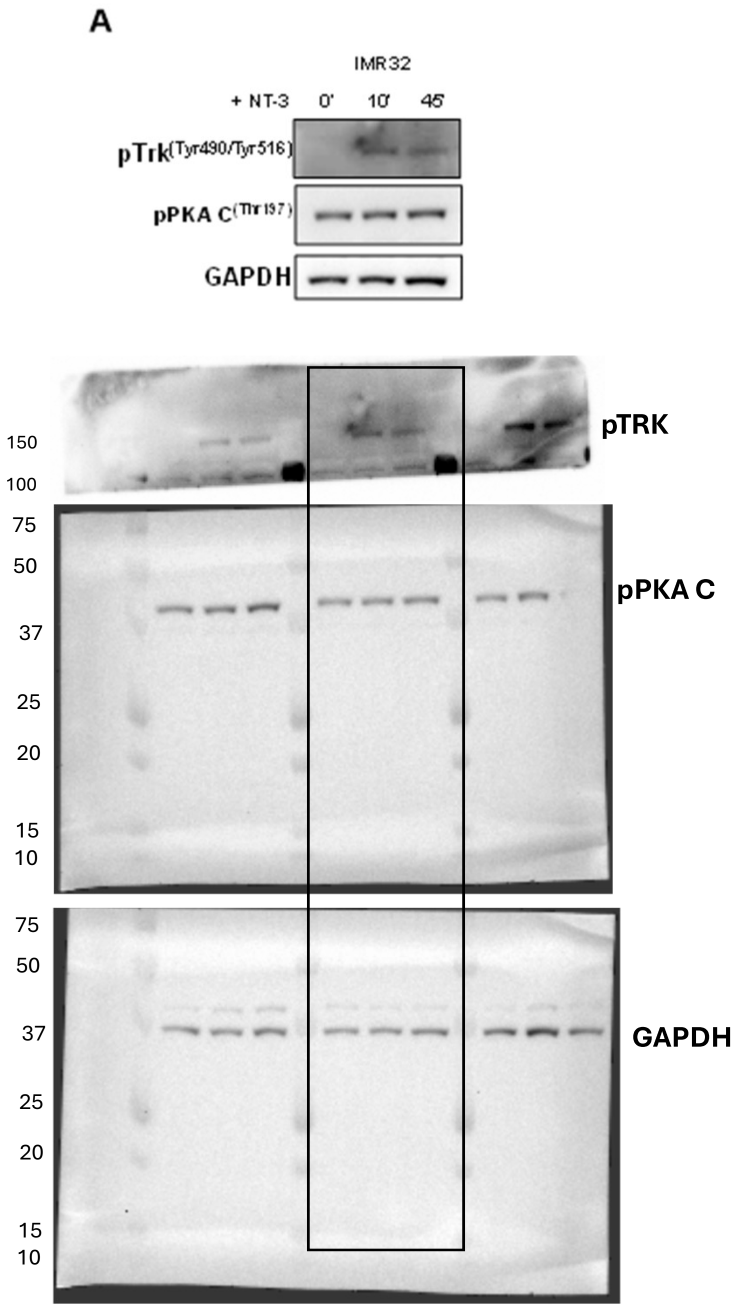

Original blots from Figure 7

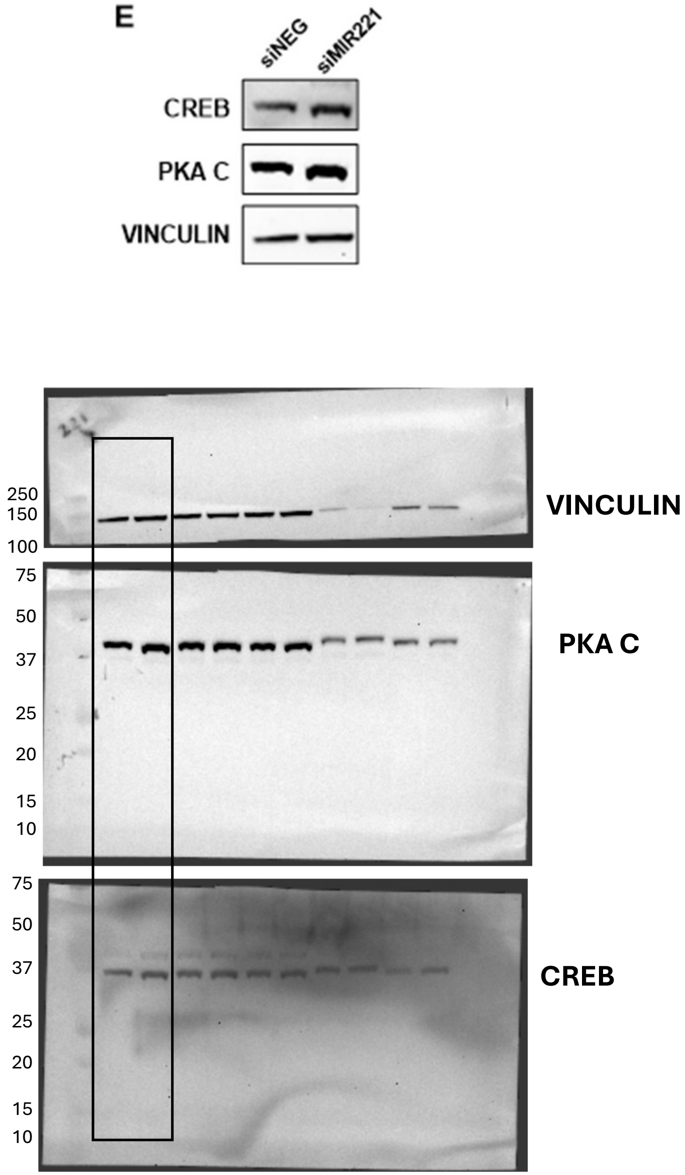

FIGURE 3

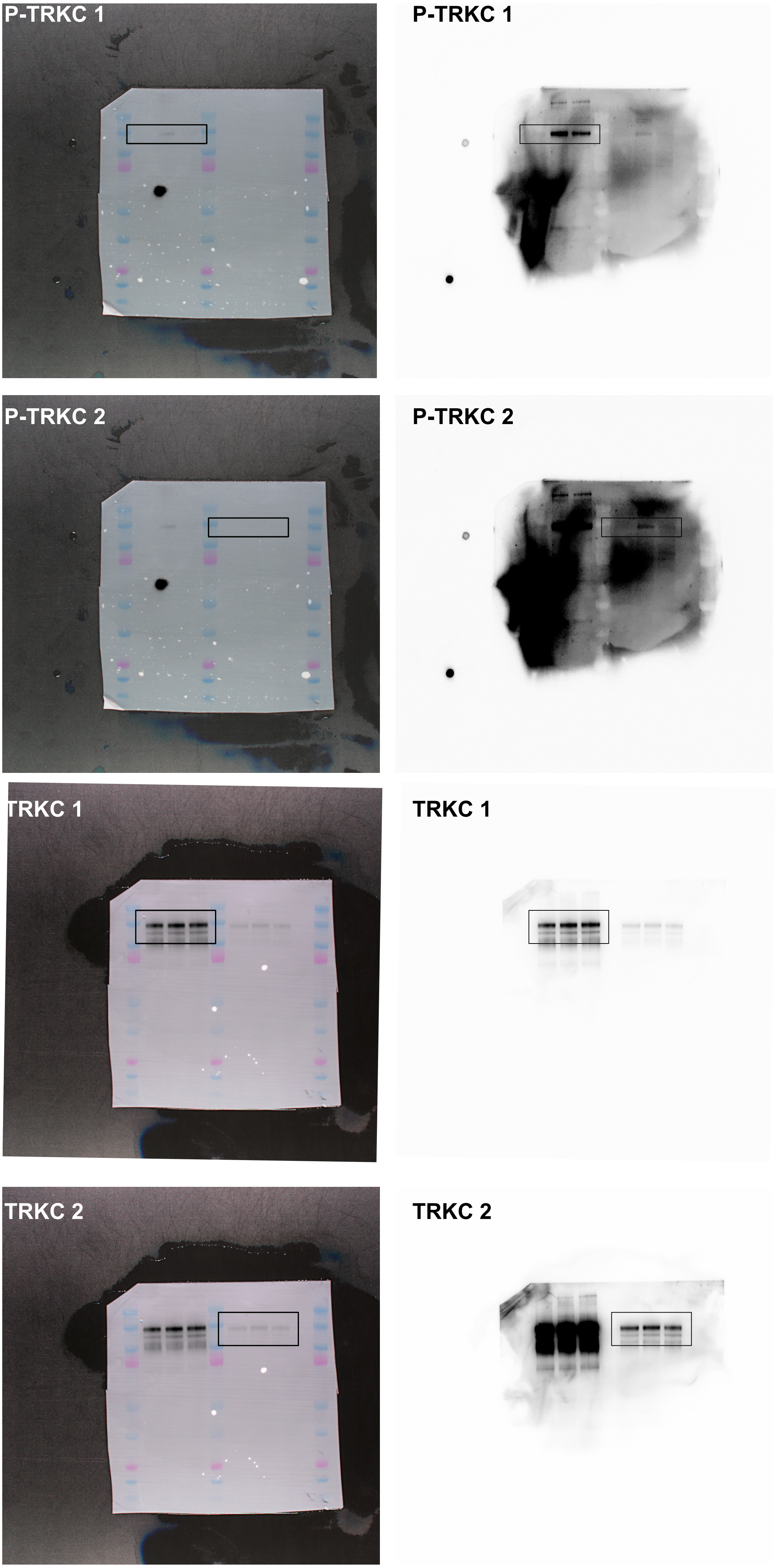

FIGURE 3

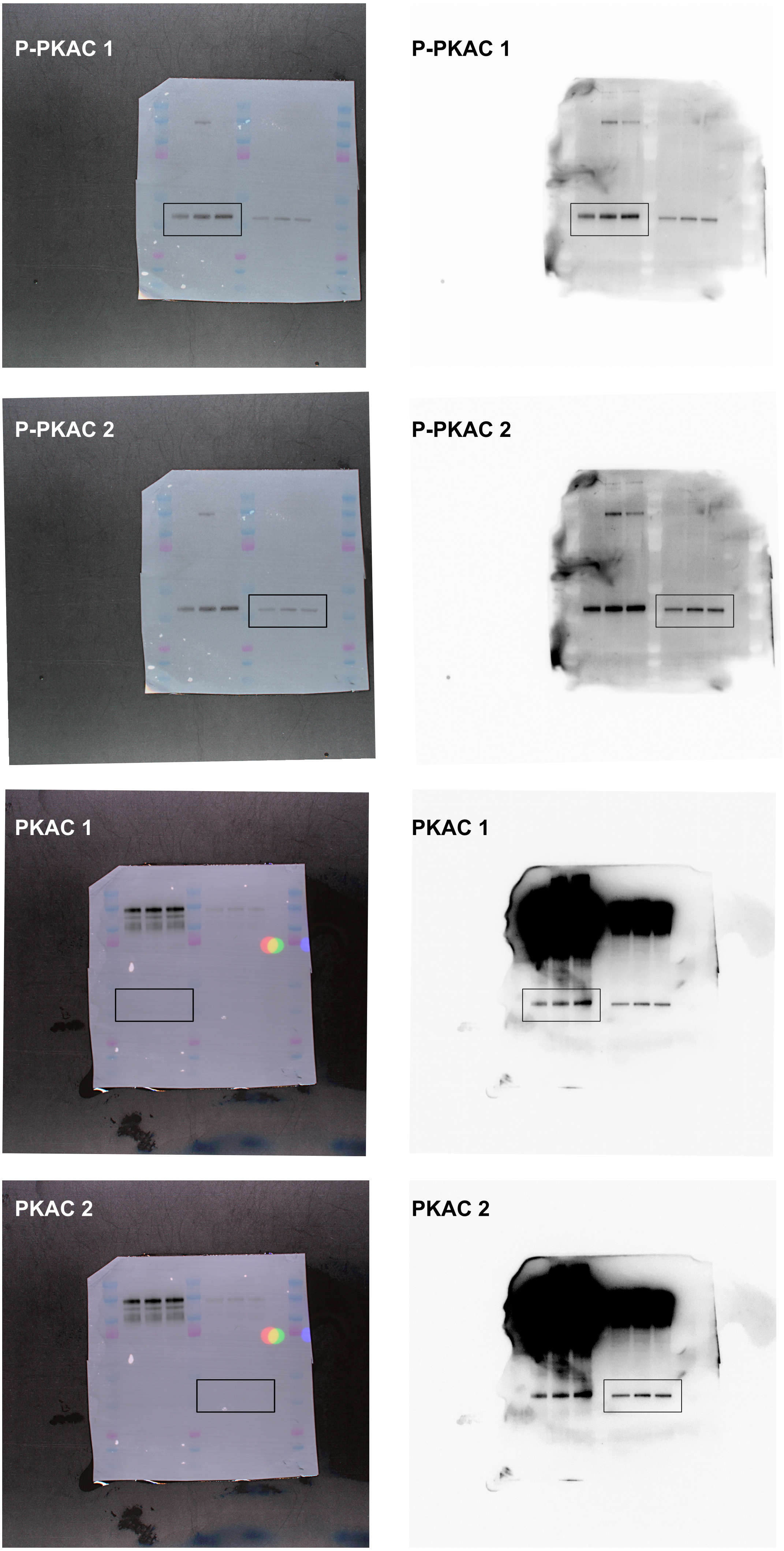

FIGURE 3

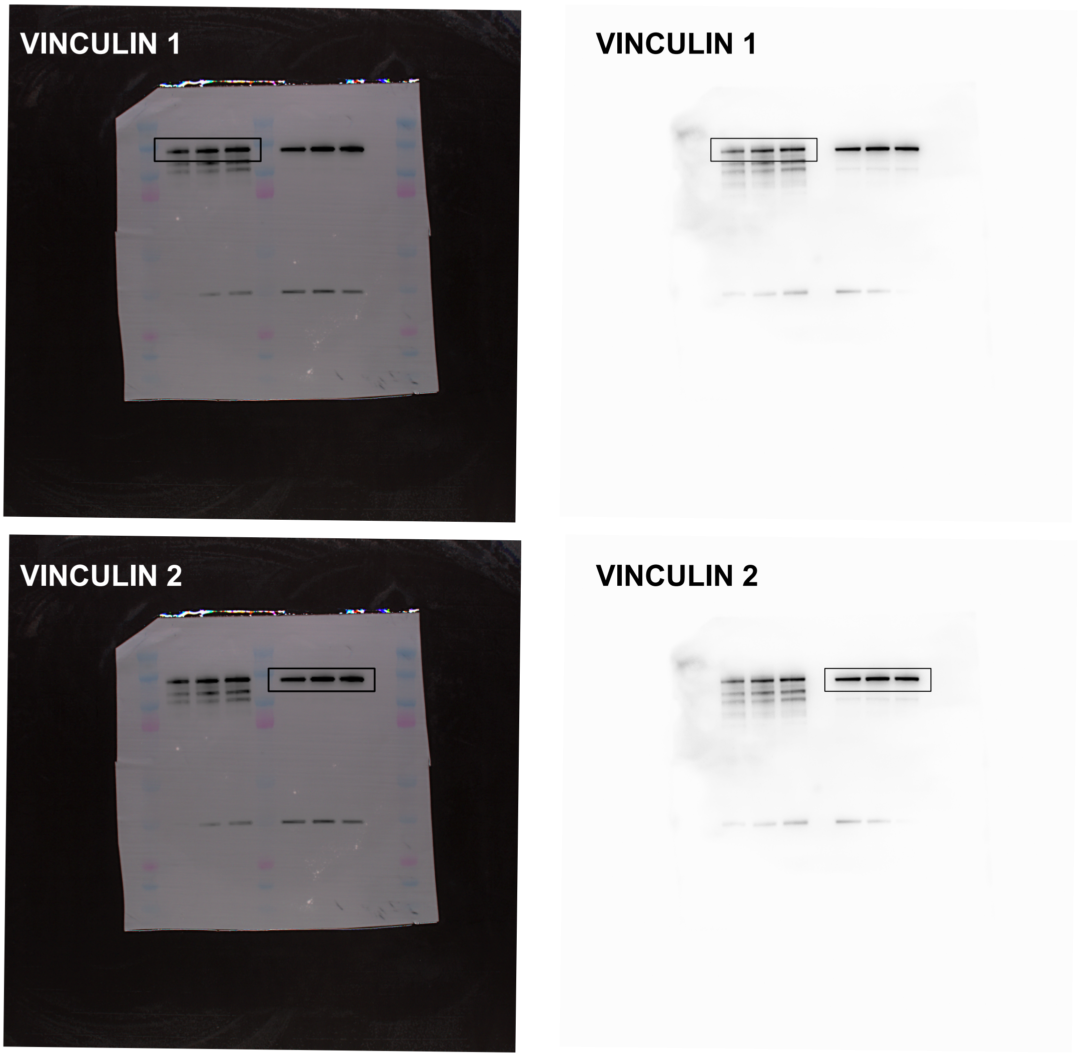

FIGURE 4

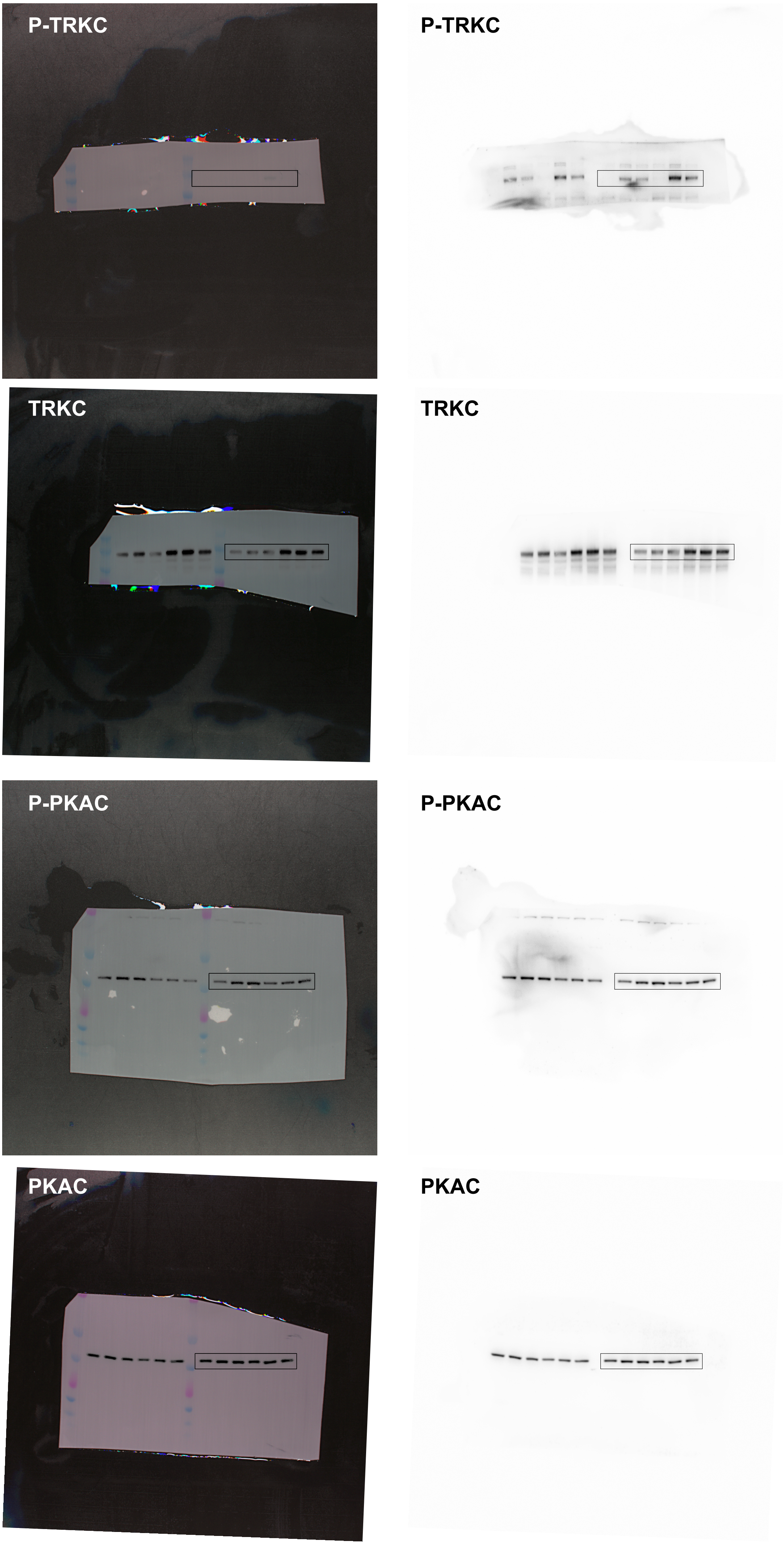

FIGURE 4

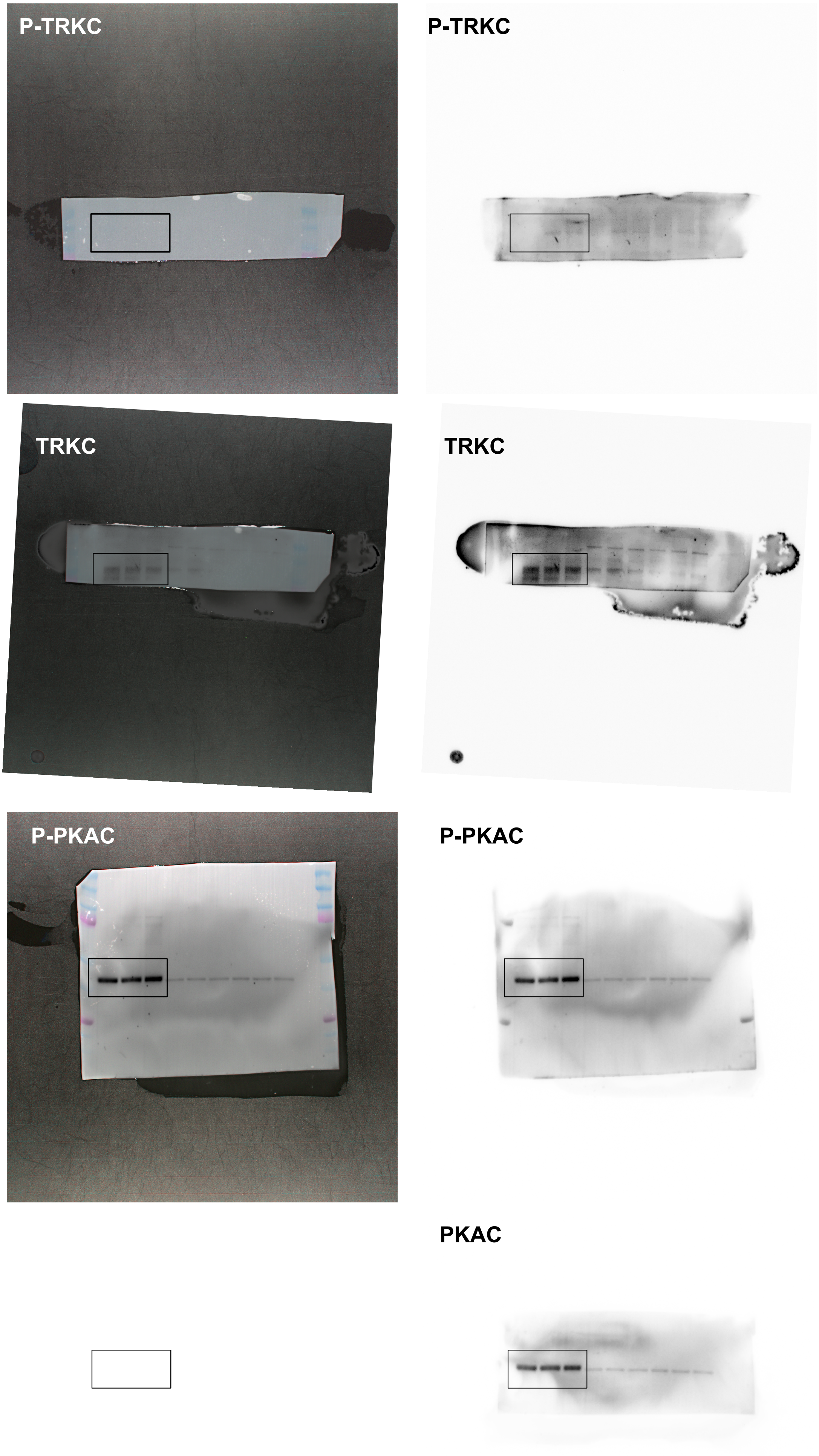

FIGURE 4

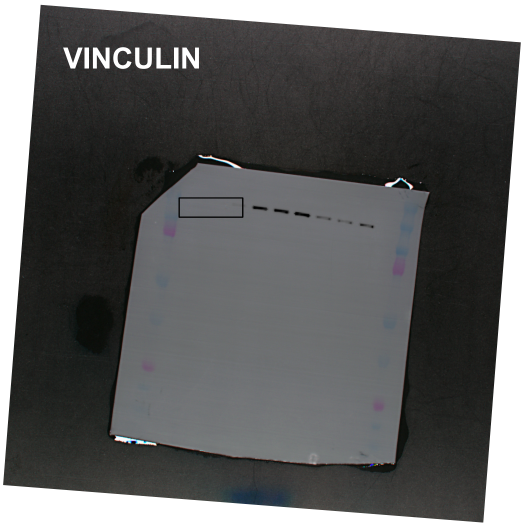

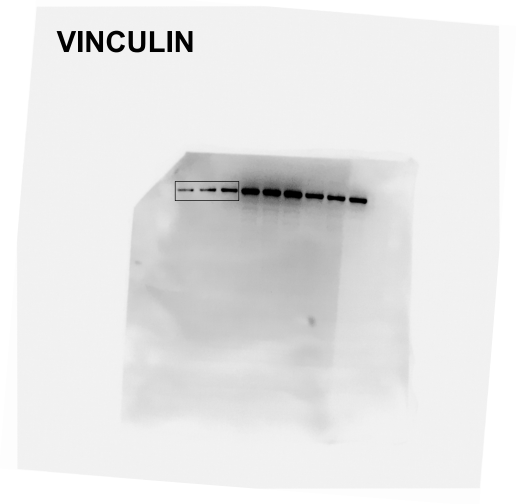

FIGURE 4

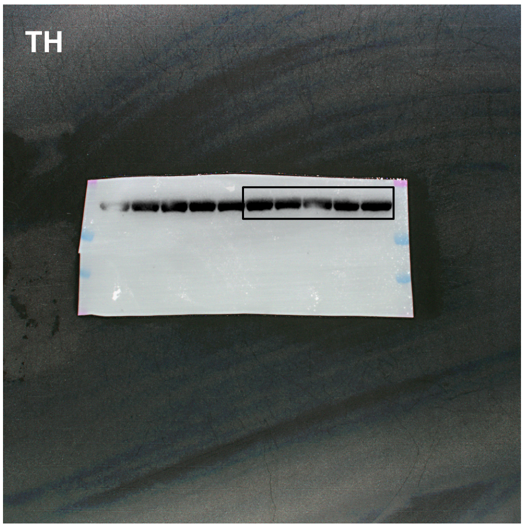

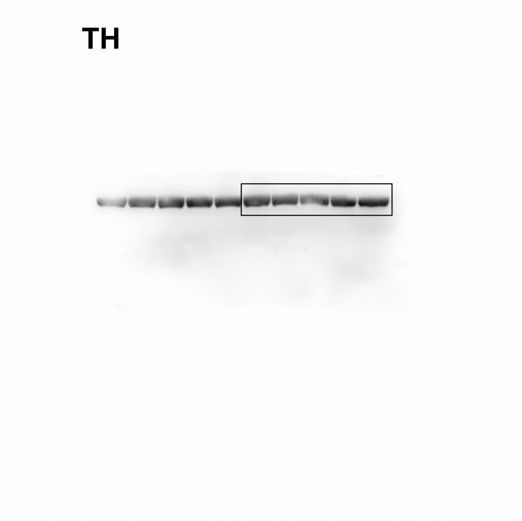

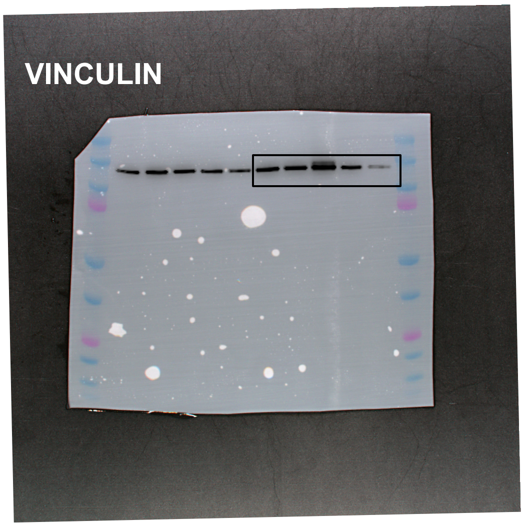

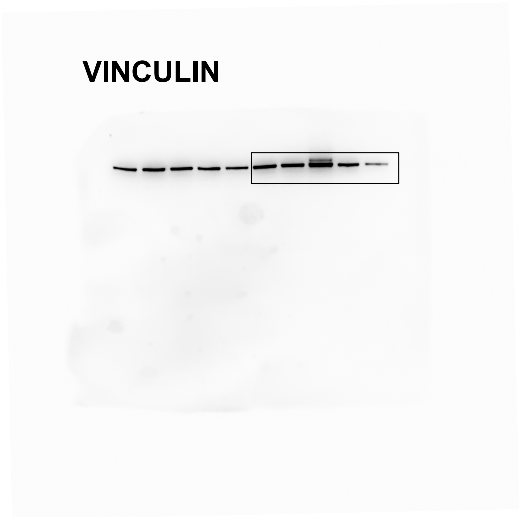

FIGURE 5

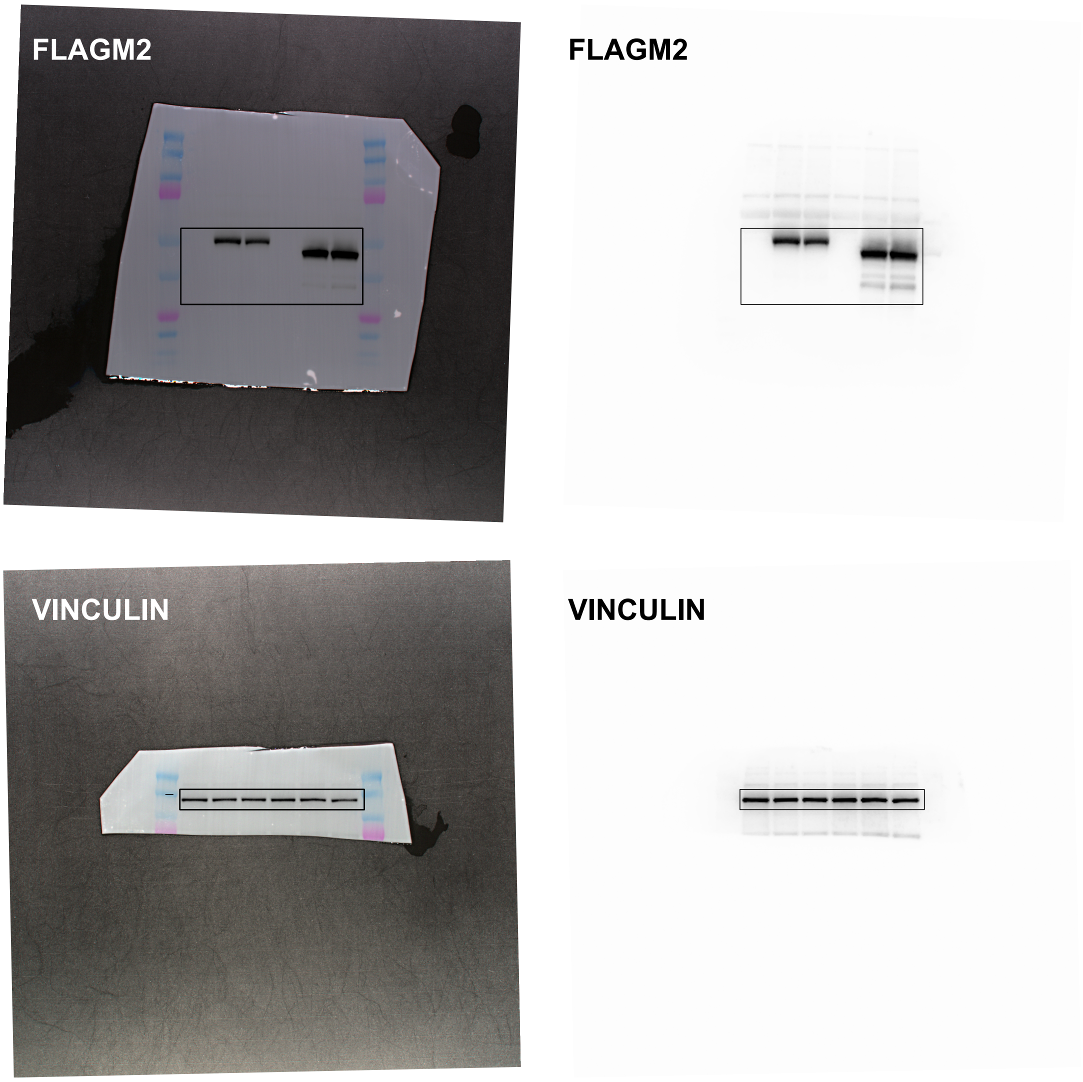

FIGURE 5

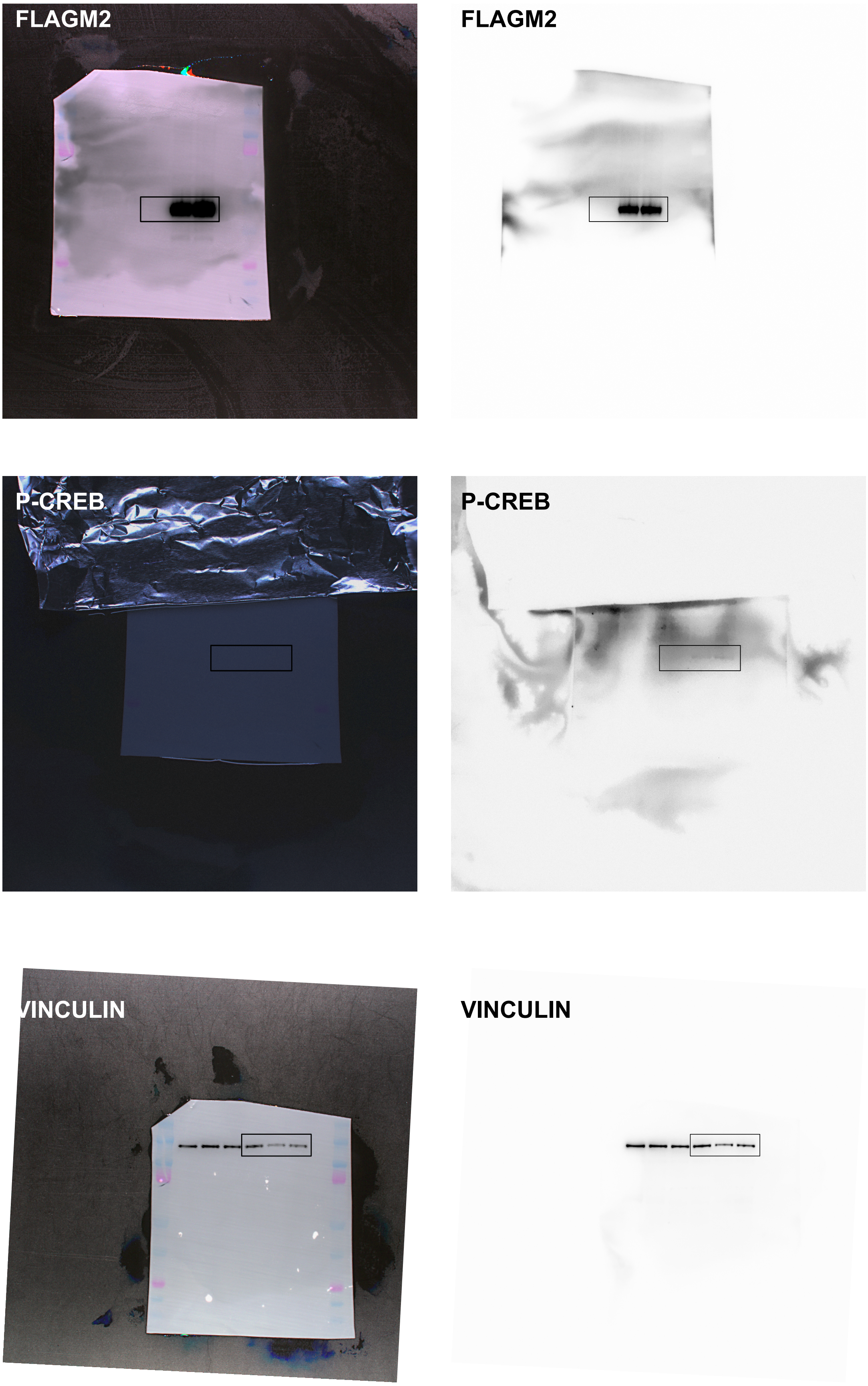

Supplementary S3

AKT

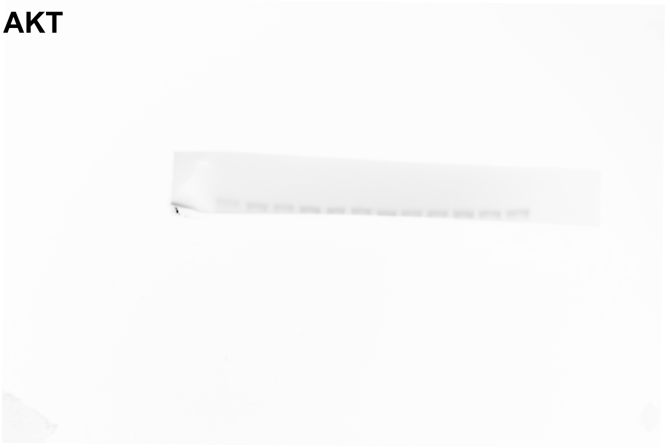

AKT

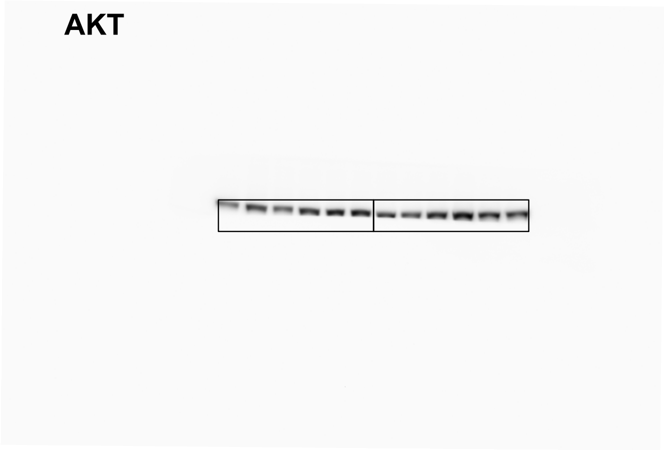

GSKB

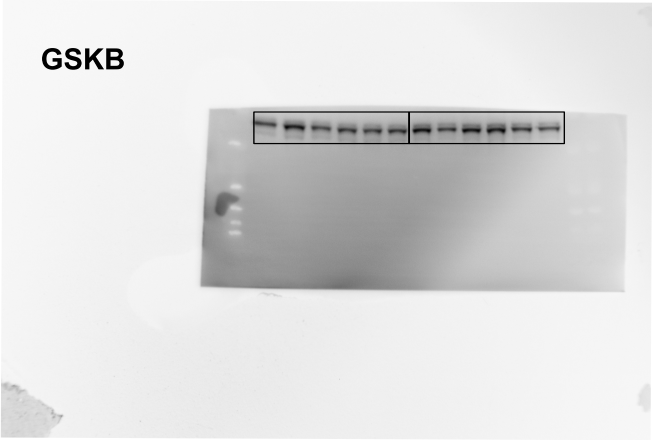

GSKB

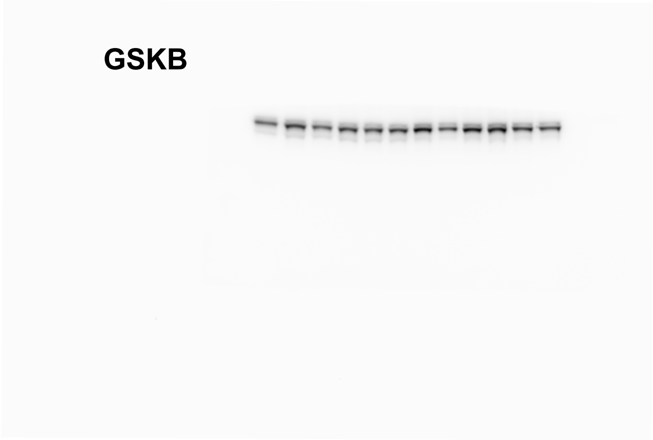

VINCULIN

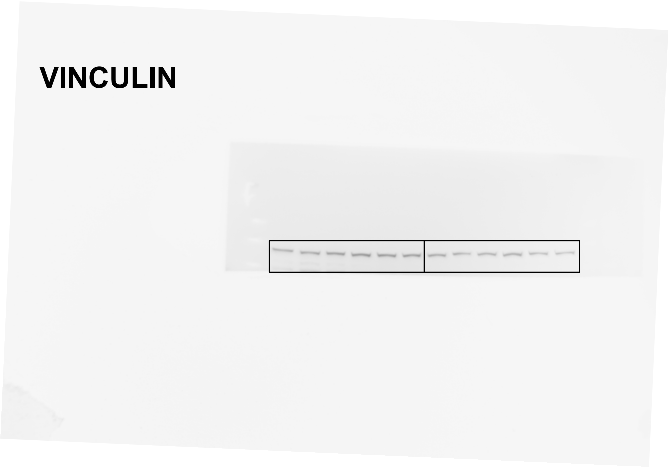

VINCULIN

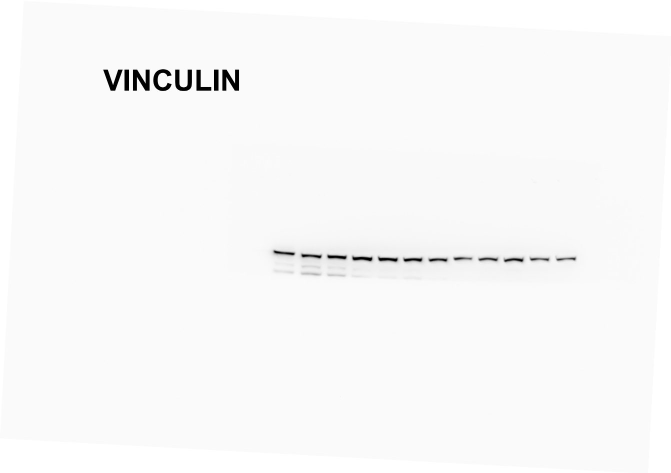

Supplement: Supplementary file 2 — Supplemental Western blots [file 41420_2026_3024_MOESM2_ESM.pdf]
